# Single-cell technology reveals the crosstalk between tumor cells and immune cells: driving immune signal transduction and inflammation-mediated cardiac dysfunction in the tumor microenvironment of colorectal cancer

**DOI:** 10.3389/fimmu.2025.1637144

**Published:** 2025-08-06

**Authors:** Wenyang Nie, Wangzheqi Zhang, Zhikai Xiahou, Yuxuan Meng, Yuhang Liu, Jingwen Zhang, Zhen Wang, Yong Wang

**Affiliations:** ^1^ First Clinical Medical College, Shandong University of Traditional Chinese Medicine, Jinan, China; ^2^ Naval Medical University, Shanghai, China; ^3^ China Institute of Sport and Health Science, Beijing Sport University, Beijing, China; ^4^ Department of Cardiovascular Diseases, Affiliated Hospital of Shandong University of Traditional Chinese Medicine, Jinan, China

**Keywords:** colorectal cancer, single-cell RNA sequencing, tumor microenvironment, immune signal transduction, cancer model, inflammation, *HMGA1*

## Abstract

**Background:**

Colorectal cancer (CRC) is a heterogeneous illness influenced by intricate tumor-immune interactions and characterized by a dismal prognosis. Macrophage-mediated immunological signaling facilitates tumor proliferation and may associate inflammation in the tumor microenvironment (TME) of CRC with negative outcomes. Notwithstanding therapeutic advancements, resistance to treatment remains a significant obstacle. scRNA-seq offers comprehensive insights into the immune signaling network and immunological dynamics inside the CRC’s TME.

**Methods:**

We integrated scRNA-seq data from GEO with extensive RNA-seq data from TCGA to elucidate immunological signaling and dynamic cellular variation in the TME of CRC. The analyses encompassed quality control via Seurat, InferCNV, Monocle, CellChat, and SCENIC, differential gene expression, inference of copy number variation (CNV), pseudo time trajectories, and intercellular communication. Prognostic modeling was conducted using Cox regression and LASSO. Immune infiltration and drug sensitivity were evaluated by CIBERSORT, ESTIMATE, xCell, TIDE, and pRRophetic. Functional validation encompassed siRNA knockdown, qRT-PCR, Western blot analysis, and cellular assessment in CRC cell lines.

**Results:**

We discovered four categories of tumor cells exhibiting variations in cell cycle, stemness, and differentiation. The *MKI67* ^+^ subpopulation exhibited a heightened dynamic cell state and engaged with macrophages via the MIF-(CD74+CD44) axis to facilitate immunological signaling. *HMGA1* is a crucial transcription factor in this fraction, and its knockdown impedes CRC cell proliferation, motility, and invasion. The cancer model utilizing the *MKI67* ^+^ TCs subpopulation (MTRS) successfully classified patient survival and linked with immune infiltration patterns and medication responses. Enrichment analysis revealed tumor-promoting and immunological signaling networks. Correlation scores suggest that this subpopulation may be linked to inflammation and immunosuppression inside the TME.

**Conclusion:**

Our research indicates that the C2 *MKI67* ^+^ TCs subpopulation is a key driver of immune signal transduction in CRC TME, which may induce inflammatory responses through interaction with macrophages, thereby leading to adverse consequences such as cardiac dysfunction. *HMGA1* represents a viable target for immunotherapy, and our cancer model derived from this subpopulation offers prognostic significance and direction for immunotherapeutic treatments.

## Introduction

Colorectal cancer (CRC) is one of the most common types of cancer in the world. It is the third most common type of cancer and the second most common cause of cancer-related death (1). GLOBOCAN 2020 statistics indicates that over 1.9 million new colorectal cancer cases are identified globally each year, resulting in more than 930,000 fatalities related to the disease (2). Notwithstanding considerable progress in early detection, surgical methods, and treatment approaches, the clinical outlook for CRC continues to pose a substantial challenge due to its heterogeneity, intricate molecular pathways, and the incidence of tumor metastasis (3, 4).

The tumor microenvironment (TME) has become a critical area of interest in comprehending tumor biology, as it plays a central role in regulating tumor growth, metastasis, and response to therapy (5–7). The TME is a multifaceted system comprising several cellular and acellular elements, such as immune cells, endothelial cells, cancer-associated fibroblasts, and cytokines (8, 9). The interaction between several stromal components in the TME and cancer cells is crucial in modulating tumor proliferation and metastasis (10). Innate and adaptive immune cells are vital components of the TME, including macrophages, neutrophils, natural killer cells, dendritic cells, T lymphocytes, and B lymphocytes, all of which actively engage in tumor genesis and progression. Macrophages are one of the most prevalent immune cell types in the TME and are typically designated as tumor-associated macrophages (TAMs) (11). Research has shown that tumor-associated macrophages facilitate colorectal cancer proliferation, metastasis, and resistance to therapy. In response to tumor-derived stimuli, TAMs move to the tumor site and polarize into either M1 or M2 phenotypes (12). Cytokines, chemokines, and growth factors released by tumor-associated macrophages influence pro-inflammatory leukocytes, endothelial cells, and fibroblasts, ultimately creating a tumor-promoting inflammatory milieu (13). Chronic inflammation is a prevalent underlying factor connecting CRC and cardiovascular diseases (CVD), which possess multiple shared risk factors. Numerous paths linking specific risk factors to cancer or cardiovascular disease converge on inflammation, whether directly or indirectly (14). A meta-analysis demonstrated a substantial correlation between ischemic heart disease (IHD) and colorectal cancers, with obesity and chronic inflammation generated by visceral fat identified as primary processes influencing both CRC and IHD (15). Researchers have been studying how tumor cells and immune cells interact more and more over the past few years. They have found that T-cell depletion, macrophage polarization, and tumor-related immunosuppressive pathways are some of the most important ways that tumors might avoid the immune system. For instance, tumor cells can change the immunological microenvironment by making PD-L1 or releasing IL-10 and TGF-β, which limit T cell activation. At the same time, immune cells like TAMs can also help tumors grow by using different signaling pathways, such as CCL2/CCR2 and CSF1/CSF1R (16). In CRC, tumor cells may engage with immune cells to elicit inflammation, consequently facilitating tumor development and contributing to heart dysfunction. A comprehensive understanding of CRC pathogenesis must encompass immune cell invasion, immunological defense, immune surveillance, and immune homeostasis (17). CRC demonstrates significant immunological heterogeneity. Certain patients, particularly those with mismatch repair deficit or elevated microsatellite instability, exhibit positive responses to immune checkpoint inhibitors such as anti-PD-1/PD-L1 treatments. Nonetheless, most microsatellite stable (MSS) patients get minimal advantage from these therapy (18).

CRC demonstrates significant variability across several tumor stages. Stages I and II are typically marked by the lack of regional lymph node metastases. Stage III is characterized by the emergence of lymph node involvement, along with heightened biological invasiveness. This stage frequently includes histological subtypes like poorly differentiated carcinoma, mucinous adenocarcinoma, or signet ring cell carcinoma, along with potential vascular, neural, and lymphatic invasion. Stage IV is characterized by distant metastases; the majority of patients exhibit a significant tumor burden and systemic symptoms such as cachexia and weight loss. Histologically, these tumors are often moderately to poorly differentiated or undifferentiated (19–21). Despite advancements in treatment modalities, including surgery, chemotherapy, and targeted medicines like anti-VEGF and anti-EGFR antibodies, the 5-year survival rate for metastatic CRC remains under 15% (22). Furthermore, medication resistance, elevated recurrence rates, and immune evasion persist as significant obstacles to existing therapeutic approaches (23, 24).

It is well known that significant advancements and innovations in the field of life sciences, such as single-cell RNA sequencing (scRNA-seq), have greatly facilitated the in-depth analysis of cellular heterogeneity and communication networks within tumor tissues of various cancer types. At the same time, machine learning and other computational methods are increasingly being utilized to discover new biomarkers and predict molecular subtypes, thereby further enhancing the translational potential of these data (25, 26). These advancements provide a theoretical basis for individualized diagnosis and treatment and are crucial instruments for comprehending cellular variation (27, 28). Furthermore, cutting-edge techniques such as scRNA-seq visualize immune signaling pathways through dynamic cellular state changes and intercellular communication, potentially enhancing the therapeutic efficacy for CRC. Consequently, we intend to elucidate a more comprehensive immune signaling network for CRC by integrating the results of multi-omics approaches, which will aid in identifying novel immunotherapeutic targets for CRC. In this study, scRNA-seq data of CRC were obtained from public sources and samples were classified according to different tumor stages to annotate and identify different tumor cell subpopulations (reclassification after subsequent difference screening). A thorough visual analysis based on single-cell properties, transcription patterns, stemness, and differentiation capacity was performed to identify essential cell subpopulations. Our study concentrated on the interactions between key subpopulations and immune-related cells, particularly macrophages, to illuminate the immune signaling of CRC within the TME and the unique responses of macrophages to tumor cells. Meanwhile, previous studies have shown that advanced CRC may affect cardiac function through immunosuppression and induction of inflammation. Therefore, we wanted to use the cardiac dysfunction (heart failure, myocardial fibrosis) score to reveal another perspective of inflammatory and immune signaling in the TME of CRC. In addition, genes or transcription factors associated with key tumor cell subpopulations were experimentally validated *in vitro*. A cancer prediction model for CRC was developed using basic subpopulation features and a comprehensive analysis of the immune infiltration landscape was performed. This work aims to provide new insights into novel targets for CRC immunotherapy and to provide possible single-cell evidence of immune signaling networks and dynamic cell state changes in the TME of CRC.

## Methods

### Data acquisition and processing

This study utilized scRNA-seq data sourced from the Gene Expression Omnibus (GEO) database (https://www.ncbi.nlm.nih.gov/geo/) with accession number GSE166555. The Cancer Genome Atlas (TCGA) portal (https://portal.gdc.cancer.gov/) was used to acquire bulk RNA-seq data. We imported the 10x Genomics data from each sample into R program (v4.3.3) via the Seurat package (v4.3.0). Initially, possible doublets and substandard cells were eliminated utilizing the DoubletFinder algorithm (v2.0.3). Cells were preserved for subsequent studies if they satisfied the following criteria: 500 < nFeature_RNA < 6000 and mitochondrial gene expression being less than 25% of total expression.

### Visualization of differentially expressed genes and AUCell-based enrichment analysis

The FindAllMarkers program (29, 30) employed the Wilcoxon rank-sum test with default parameters (log fold change > 0.25) to find differentially expressed genes (DEGs) for each cell type and subpopulation. Enrichment analyses were conducted utilizing the clusterProfiler (v4.6.2) and Single Cell Profiler (SCP) (v0.4.8) packages to clarify the functional roles of DEGs within each cell type and subpopulation. All enrichment pathways were obtained from Gene Ontology (GO) (29–34). Furthermore, we utilized AUCell (35, 36) to discern active gene sets and transcription factors at the single-cell resolution.

### Inference of copy number variation levels

The InferCNV method (v1.17.0) was employed to ascertain copy number variation (CNV) levels (37). Copy number karyotyping of aneuploid cells was employed during carcinogenesis to distinguish between non-malignant and malignant tumor cells. Endothelial cells served as the reference population for CNV comparison to ascertain whether other tumor cells displayed significant chromosomal copy number variations.

### Construction of pseudotime trajectories

Pseudotime trajectories of CRC tumor cell subpopulations were recreated utilizing the Monocle software (v2.24.0), based on single-cell RNA sequencing data. This methodology simulates the dynamic evolution of individual cells and elucidates the transcriptional alterations linked to the differentiation of tumor cells in CRC progression.

### CytoTRACE and slingshot analyses

We utilized CytoTRACE to analyze variations in developmental and differentiation states among CRC tumor cell subpopulations, inferring and ranking the differentiation capacity of all tumor cell clusters. Furthermore, the getlineage and getCurves functions were employed to deduce lineage trajectories and evaluate dynamic alterations in gene expression during pseudotime. The Slingshot software (v2.6.0) was utilized to generate developmental trajectories, offering insights into the differentiation status and advancement of each tumor cell subpopulation (38).

### CytoTRACE2 evaluation

We utilized CytoTRACE2 for a comprehensive analysis of the scRNA-seq data, with the objective of predicting the potential subtypes and absolute developmental capacities of tumor cell subpopulations originating from tumors at various clinical stages. In CytoTRACE2, latent classes are delineated according to cellular developmental potential, offering a continuous spectrum from 0 (completely differentiated) to 1 (totipotent) to quantify developmental capacity. This facilitates direct comparisons among datasets in absolute developmental space. CytoTRACE primarily relies on gene expression diversity, while CytoTRACE2 employs an integrated model that combines multiple feature-optimized approaches. We therefore believe that the two methods are complementary, which helps enhance the stability and reliability of the inferred results. Moreover, CytoTRACE2 enables the detection and validation of differentiation status among all tumor cell subpopulations.

### Analysis of cell–cell communication

Utilizing scRNA-seq data, we employed the CellChat software (v1.6.1) to forecast probable intercellular interactions among several cell types, encompassing tumor cell subpopulations and additional stromal or immune cells. Ligand-receptor interactions were deduced with CellChatDB.human as the reference database. A significance level of P < 0.05 was utilized to discern substantial interactions across different cell types or tumor subpopulations. This research concentrated on visualizing and analyzing the interactions between major tumor subpopulations and relevant immune cells, such as macrophages and mast cells, to identify potential signaling pathways of relevance.

### SCENIC evaluation

We conducted SCENIC analysis to reconstruct gene regulation networks from scRNA-seq data and to identify stable cellular states. Specifically, we utilized the pySCENIC module (v0.12.1) in Python (v3.9.19) to infer transcription factor activity and generate an AUCell matrix for evaluating regulon enrichment and activity. The outcomes were later visualized using R software (v4.3.3) to enhance the understanding of transcriptional regulation among cell groups.

### Development of a prognostic model utilizing CRC tumor cells

We evaluated the prognostic significance of essential tumor cell subpopulations as survival indicators in colorectal cancer by utilizing their marker genes as potential predictive attributes. The survival R package was utilized to perform univariate Cox regression and LASSO regression studies to identify additional genes pertinent to prognosis. Using a multivariate Cox regression model, a prognostic signature was created, and the formula was applied to calculate each patient’s risk score: Risk score = (gene1 expression × coefficient1) + (gene2 expression × coefficient2) +… + (geneN expression × coefficientN).

Based on the median risk score, patients were divided into high-risk and low-risk groups, with scores above the median considered high risk and those below considered low risk. We conducted a Kaplan–Meier survival analysis to investigate the disparities in overall survival (OS) across the groups (39). To evaluate the model’s predictive accuracy and calibration, the timeROC package (v0.4) was employed to produce time-dependent ROC curves for 1, 3, and 5 years. A multivariate Cox regression analysis was performed to determine whether the risk score functioned as an independent prognostic factor. A nomogram was created to predict OS at 1, 3, and 5 years, with internal validation conducted through the concordance index (C-index) and calibration plots.

### Survival analysis

Transcriptomic data from colorectal cancer patients, together with extensive clinical information, were obtained from The Cancer Genome Atlas (TCGA) database (https://portal.gdc.cancer.gov/) for subsequent study. Patients were categorized into two groups according to the expression levels of specific genes: the high *MKI67^+^* TCs risk score group (High MTRS group) and the low *MKI67^+^* TCs risk score group (Low MTRS group). Kaplan–Meier survival curves were constructed using the survival program (v3.5-5) and displayed with the survminer tool (v0.4.9) to assess survival outcome disparities between these groups.

### Analysis of immune infiltration

We assessed the invasion of 22 immune cell types utilizing the CIBERSORT R program (v0.1.0). Following this, the CIBERSORT, ESTIMATE, and xCell algorithms were employed to evaluate the immune microenvironment in CRC patients, including differences in immune cell infiltration levels and the varied expression of genes related to immune checkpoints. Visual tools were created to show the relationships between immune cells, model genes, OS, and risk scores. The Tumor Immune Dysfunction and Exclusion (TIDE) platform (http://tide.dfci.harvard.edu) was utilized to forecast patients’ responses to immunotherapy. The forecasts of drug immune responses were then validated utilizing data from the TCIA database (https://www.cancerimagingarchive.net/).

### Pharmacological sensitivity assessment

Our study included therapeutically pertinent medications to assess drug sensitivity among patient cohorts. pRRophetic program (v0.5) was employed to determine the IC50 for each drug. To assess differences in drug sensitivity, the projected IC50 values for high-risk and low-risk groups were compared.

### Cellular cultivation

Procell Life Science & Technology Co., Ltd. (Wuhan, China) supplied the HCT116 and HT-29 human colorectal carcinoma cell lines. The cells were cultured in MEM supplemented with 10% fetal bovine serum (FBS), 100 U/mL penicillin, and 0.1 mg/mL streptomycin. All cultures were preserved in a humidified incubator at 37°C with 5% CO_2_ concentration. Cells in the logarithmic growth phase were harvested for following experimental procedures.

### siRNA transfection

Cells were seeded in 6-well plates at a density of 2 × 10^5^ cells per well for transfection assays. Following a 24-hour incubation period, cells were transfected with *HMGA1*-targeting small interfering RNAs (siRNAs) obtained from GenePharma (Shanghai, China) at a final concentration of 20 µM. Transfection was performed via Lipofectamine RNAiMAX (Life Technologies, Thermo Fisher Scientific, Brendale, QLD, Australia) in accordance with the manufacturer’s instructions. Cells were collected 24 hours after transfection for further analysis. The sequences of the *HMGA1* siRNAs employed were as follows: siRNA1, ACUCCAGGAAGGAAACCAA; siRNA2, AGCGAAGUGCCAACACCUA.

### RNA isolation and real-time quantitative polymerase chain reaction

Total RNA was extracted utilizing TRIzol reagent (Thermo Fisher Scientific, Waltham, MA, USA) in accordance with the manufacturer’s guidelines. Subsequently, 500 ng of total RNA was reverse transcribed into cDNA utilizing the PrimeScript™ RT Reagent Kit (TaKaRa, Tokyo, Japan). Quantitative real-time PCR (qRT-PCR) was conducted with SYBR^®^ Premix Ex Taq™ (TaKaRa) on an ABI ViiA™ 7 Real-Time PCR System (Applied Biosystems, Indianapolis, IN, USA) (40). The primers for *HMGA1* were specifically designed as follows: Forward primer: 5′-AGTGAGTCGAGCTCGAAGTC-3′; reverse primer: 5′-GTCTCTTAGGTGTTGGCACT-3′.

### Cell viability assessment

The Cell Counting Kit-8 (CCK-8; Dojindo Laboratories, Kumamoto, Japan) was employed to assess cell viability. The cells were inoculated into 96-well plates at a density of 1,000 cells per well and incubated overnight. Subsequently, 100 µL of CCK-8 working solution was added to each well and incubated for 1 hour at 37°C. The optical density at 450 nm was measured daily for four consecutive days utilizing a microplate reader. Cell growth curves were constructed by graphing OD_450_ values over time to assess cell viability and proliferation.

### Clonal formation assay

Cells in the logarithmic growth phase were collected, resuspended, and diluted to the specified concentration. One thousand cells per well were inoculated into 6-well plates and cultivated for ten days under conventional conditions, with periodic inspection. Upon the formation of visible colonies, the culture media was discarded, and the cells were meticulously washed twice with ice-cold phosphate-buffered saline (PBS). Cells were subsequently fixed with 4% paraformaldehyde for 20 minutes at ambient temperature, followed by staining with 0.1% crystal violet for 10 minutes. The quantity of colonies was assessed utilizing a gel imaging analysis equipment (G: BOX-F3EE, Syngene, Bangalore, India).

### EdU assessment

Cells in the logarithmic growth phase were collected, resuspended, and diluted accordingly. 1×10³ cells were inoculated per well in 6-well plates. The EdU incorporation test kit from RiboBio (Guangzhou, China) was utilized to evaluate cell proliferation in accordance with the manufacturer’s guidelines. Following staining, EdU-positive cells were observed utilizing a fluorescence microscope. Quantification involved counting EdU-positive cells and total cells in a minimum of six randomly chosen fields per sample.

### Wound healing assay

In 6-well plates, cells were placed at a density of 2 × 10^5^ cells per well and incubated overnight to ensure they adhere. A linear incision was made in the confluent monolayer utilizing a 10 µL pipette tip positioned perpendicularly to the plate surface. The wells were washed thrice with PBS to eliminate unattached cells, after which fresh serum-free media was introduced. The plates were thereafter incubated at 37°C in a humidified incubator containing 5% CO_2_. Wound closure was assessed by taking images at 0 and 48 hours utilizing bright-field microscopy.

### Transwell migration and invasion assay

A Transwell test was performed to evaluate cell migration and invasion. Cells were inoculated into 24-well Transwell insert chambers (BD Biosciences, USA) filled with serum-free media. For the invasion assay, the inserts were pre-treated with 2% Matrigel. The lower chamber housed a medium supplemented with 20% FBS to serve as a chemoattractant. After a 48-hour incubation period, cells adhering to the upper membrane surface were meticulously removed, while those that penetrated the membrane were fixed with paraformaldehyde and stained with crystal violet. The dyed cells were subsequently photographed and quantified using a microscope.

### Statistical analysis

Using R software (v4.3.3) and Python software (v3.9.19), statistical analysis was carried out. Wilcoxon’s test and Pearson’s correlation coefficient were used to evaluate the significance of differences between groups. The subsequent interpretation was utilized for P-values: *P < 0.05, **P < 0.01, ***P < 0.001, ****P < 0.0001. Insignificant differences were designated as ‘ns’. The statistical techniques and significance levels used evaluated the validity of the experimental findings and offered strong backing for the conclusions.

## Results

### Heterogeneity of the TME in CRC

**Figure 1:**
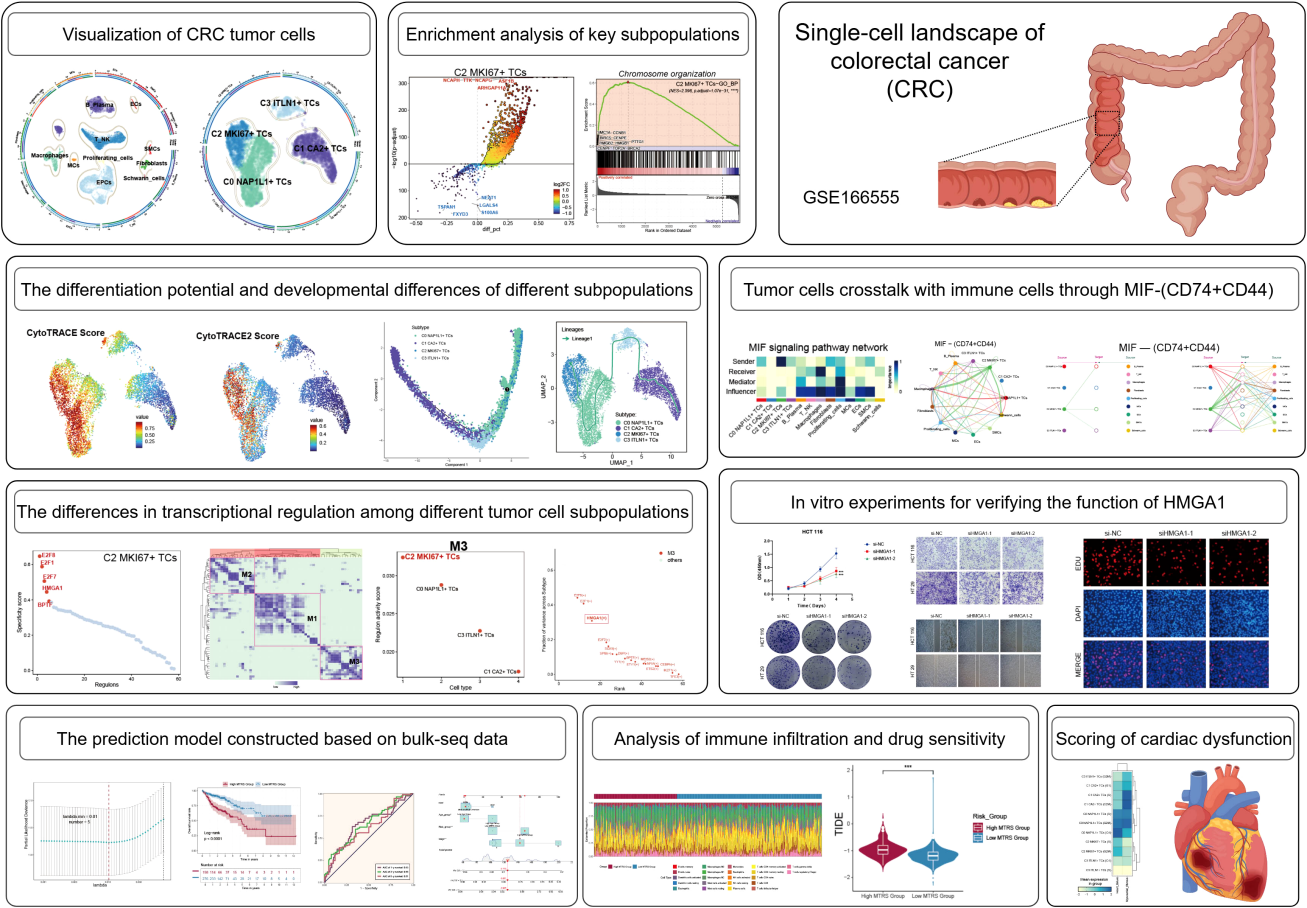
Overview of the research concepts and substance of this study.

**Figure 2:**
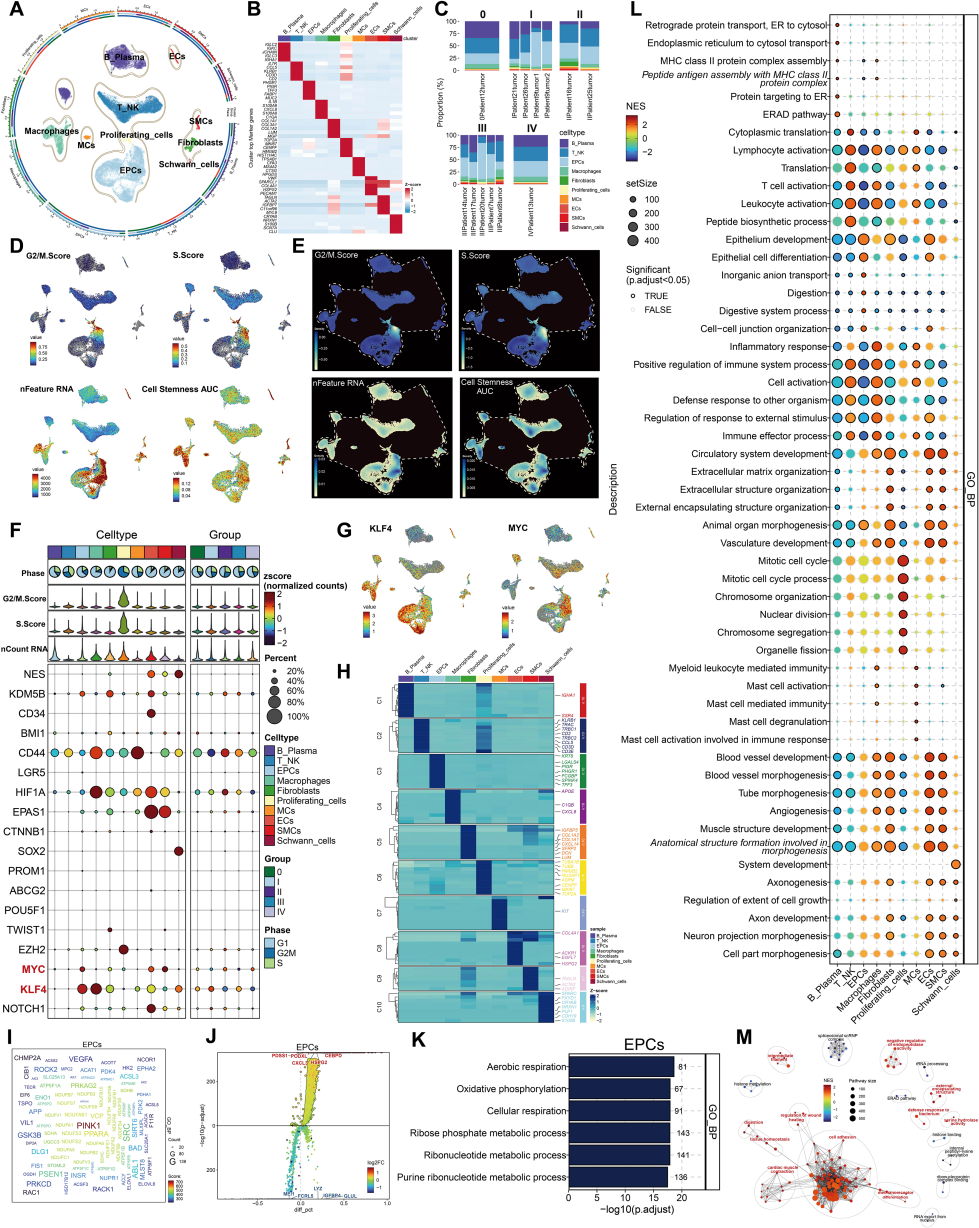
Cellular heterogeneity in CRC tissue. **(A)** All cell types were identified following dimensionality reduction, grouping, and annotation. **(B)** Five principal marker genes discovered across all cell types. **(C)** Proportions of each cell type within different tumor samples, categorized by five distinct tumor phases. **(D)** The UMAP visualization displayed the G2/M score, S score, nFeature RNA, and cell stemness AUC for all cells. **(E)** UMAP-derived relative density distributions exhibited the G2/M score, S score, nFeature RNA, and cell stemness AUC for all cells. **(F)** Expression profiles of stemness-related genes were examined across all cells and tumor stage groups. **(G)** UMAP plots revealed differential expression of the stemness genes *KLF4* and *MYC* among all cells. **(H)** The heatmap depicted differentially expressed genes among all cell groups. **(I)** The word cloud emphasized genes that exhibited a strong correlation with EPCs. **(J)** Volcano plot displayed the top 5 upregulated and top 5 downregulated genes in EPCs. **(K, L)**. GO-BP and GO-GSEA enrichment analysis were conducted using EPC-related differentially expressed genes. **(M)**. The network diagram depicted the enhanced pathways related to EPCs.

The overarching concept and substance of our investigation are illustrated in **Figure 1**. To begin with, We picked 13 colorectal cancer tumor tissue samples representing various tumor stages from the dataset. Following rigorous quality control and batch effect elimination, a total of 37,236 high-quality cells were preserved. Following dimensionality reduction and clustering investigations, ten different cell groupings were discovered. Clusters were annotated according to the differential expression of established cell-type-specific marker genes as follows: B-plasma cells, T-NK cells, endothelial progenitor cells (EPCs), macrophages, fibroblasts, proliferating cells, mast cells (MCs), endothelial cells (ECs), smooth muscle cells (SMCs), and Schwann cells (**Figure 2A**). **Figure 2B** displays the five principal marker genes for each cluster, with EPCs identified by *PHGR1*, *PIGR*, *TFF3*, *FABP1*, and *MUC2*. EPCs were primarily concentrated in tumor stages III, I, and IV (**Figure 2C**). InferCNV analysis indicated a unique CNV pattern in EPC, suggesting that it may be related to tumor origin (**Supplementary Figure 1A**). Moreover, EPCs and proliferating cells demonstrated markedly increased G2/M and S scores. EPCs exhibited the greatest nFeature_RNA counts, succeeded by macrophages, proliferating cells, ECs, fibroblasts, and SMCs. In terms of cellular stemness, macrophages, MCs, ECs, and SMCs had comparatively high scores, while EPCs ranked marginally lower although remained elevated relative to other cell types (**Figure 2D**). **Figure 2E** depicts the significant relative density of EPCs in the G2/M score, S score, and nFeature_RNA. EPCs, macrophages, MCs, and T-NK cells demonstrated elevated concentrations of cell stemness AUC. A bubble plot demonstrated that *MYC* and *KLF4* were among the most highly expressed stemness-associated genes in EPCs (**Figure 2F**). UMAP imaging validated the heightened expression of *KLF4* and *MYC* primarily within the EPC population (**Figure 2G**). To enhance the characterization of the TME and EPCs in CRC, we performed a series of enrichment analyses. The heatmap illustrated differential gene expression among all cell types identified genes like *KRT8*, *LGALS4*, *PIGR*, *PHGR1*, *FCGBP*, *SPINK4*, and *TFF3* as considerably enriched in EPCs (**Figure 2H**). *PINK1* was identified as a significantly enriched gene (**Figure 2I**). The five most upregulated genes in EPCs were *PDSS1*, *PODXL*, *CXCL3*, *HSPG2*, and *CEBPD*, while the five most downregulated genes were *MEI1*, *FCRL5*, *LYZ*, *IGFBP4*, and *GLUL* (**Figure 2J**). The GO Biological Process (GO-BP) analysis indicates that EPCs are mostly linked to aerobic respiration, oxidative phosphorylation, cellular respiration, ribose phosphate metabolism, ribonucleotide metabolism, and purine ribonucleotide metabolism (**Figure 2K**). GO-GSEA analysis additionally associated EPCs with epithelial growth, epithelial cell differentiation, inorganic anion transport, digestion, digestive system functions, cell–cell junction architecture, and organ morphogenesis in animals (**Figure 2L**). Finally, the enrichment network (**Figure 2M**) identified pathways associated with cell adhesion, cardiac muscle contraction, mechanoreceptor differentiation, tissue homeostasis, digestion, wound healing regulation, intermediate filaments, negative regulation of endopeptidase activity, external encapsulating structures, bacterial defense response, and serine hydrolase activity in relation to EPCs.

### Characterization of tumor cell subpopulations in CRC at the single-cell level

**Figure 3:**
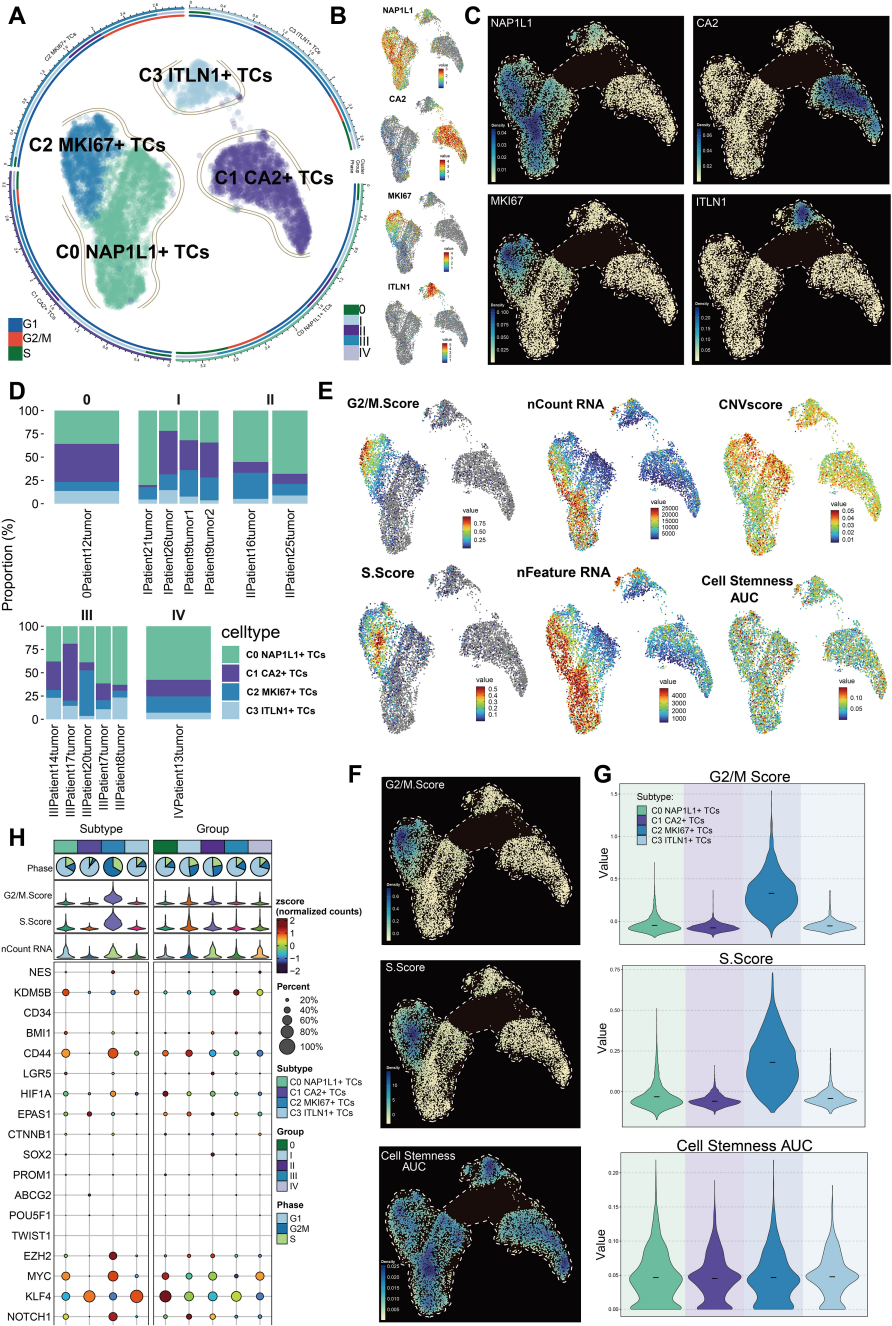
Heterogeneity of subpopulations within CRC tumor cells. **(A)** Depiction of four tumor cell subpopulations categorized by uniquely expressed marker genes, alongside the distribution of cell cycle phases and tumor stages within each subpopulation. **(B)** Expression profiles of the nomenclature genes *NAP1L1*, *CA2*, *MKI67*, and *ITLN1* among the four subpopulations. **(C)** Variations in relative density among the four designated genes across subpopulations. **(D)** Proportions of each tumor cell subpopulation within distinct tumor samples across various groups. **(E)** UMAP visualizations displayed the G2/M score, S score, nCount RNA, nFeature RNA, CNV score, and cell stemness AUC across all tumor cell subpopulations. **(F)** UMAP-based density plots indicated the relative differences in G2/M score, S score, and cell stemness AUC between subpopulations. **(G)** Violin plots contrasted the rankings of G2/M score, S score, and cell stemness AUC among subpopulations. **(H)** Variations in the expression of stemness-related genes were examined across all tumor cell subpopulations.

We classified a total of 7,759 tumor cells into separate subpopulations based on their DEGs and designated each cluster according to its most prominently expressed marker gene: C0 *NAP1L1^+^* TCs, C1 *CA2^+^* TCs, C2 *MKI67^+^* TCs, and C3 *ITLN1^+^* TCs (**Figure 3A**). The UMAP visualization revealed that the hallmark genes *NAP1L1*, *CA2*, *MKI67*, and *ITLN1* were primarily expressed in their specific subpopulations (**Figure 3B**). Moreover, these four marker genes had the greatest expression density within their respective clusters (**Figure 3C**). C0 *NAP1L1^+^* TCs comprised the predominant fraction and were significantly represented across all five tumor stages. C1 *CA2^+^* TCs exhibited greater abundance in tumor stages 0, I, and III. C2 *MKI67^+^* TCs exhibited increased proportions predominantly in stages II and III, ranking second only to C0 *NAP1L1^+^* TCs in stage IV. C3 *ITLN1^+^* TCs were less prevalent overall but exhibited a comparatively greater presence in stage III (**Figure 3D**). We subsequently analyzed the distribution of G2/M score, S score, nCount_RNA, nFeature_RNA, CNV score, and cell stemness AUC among the tumor cell subpopulations with UMAP plots (**Figure 3E**). We additionally analyzed the disparities in expression density of the G2/M score, S score, and cell stemness AUC among these clusters (**Figure 3F**). C2 *MKI67^+^* TCs demonstrated the greatest G2/M and S scores, but C0 *NAP1L1^+^* TCs and C2 *MKI67^+^* TCs displayed comparatively increased nFeature_RNA and nCount_RNA values. Nonetheless, the variations in cell stemness AUC among the subpopulations were not statistically significant. Violin plots distinctly demonstrated that the G2/M and S scores of C2 *MKI67^+^* TCs were significantly elevated compared to those of other clusters, although the difference in cell stemness AUC among subpopulations was minimal (**Figure 3G**). Despite the absence of significant variations in overall cell stemness among clusters, we continued to investigate the expression of genes associated with stemness. **Figure 3H**’s bubble plot demonstrated that C0 *NAP1L1^+^* TCs exhibited elevated levels of *KDM5B*; C1 *CA2^+^* TCs expressed *EPAS1* and *KLF4*; C2 *MKI67^+^* TCs displayed significant expression of *EZH2*, *NOTCH1*, *MYC*, and *CD44*; whereas C3 *ITLN1^+^* TCs mostly expressed *KLF4*.

### Differentiation potential and developmental stage variations of CRC tumor cell subpopulations

**Figure 4:**
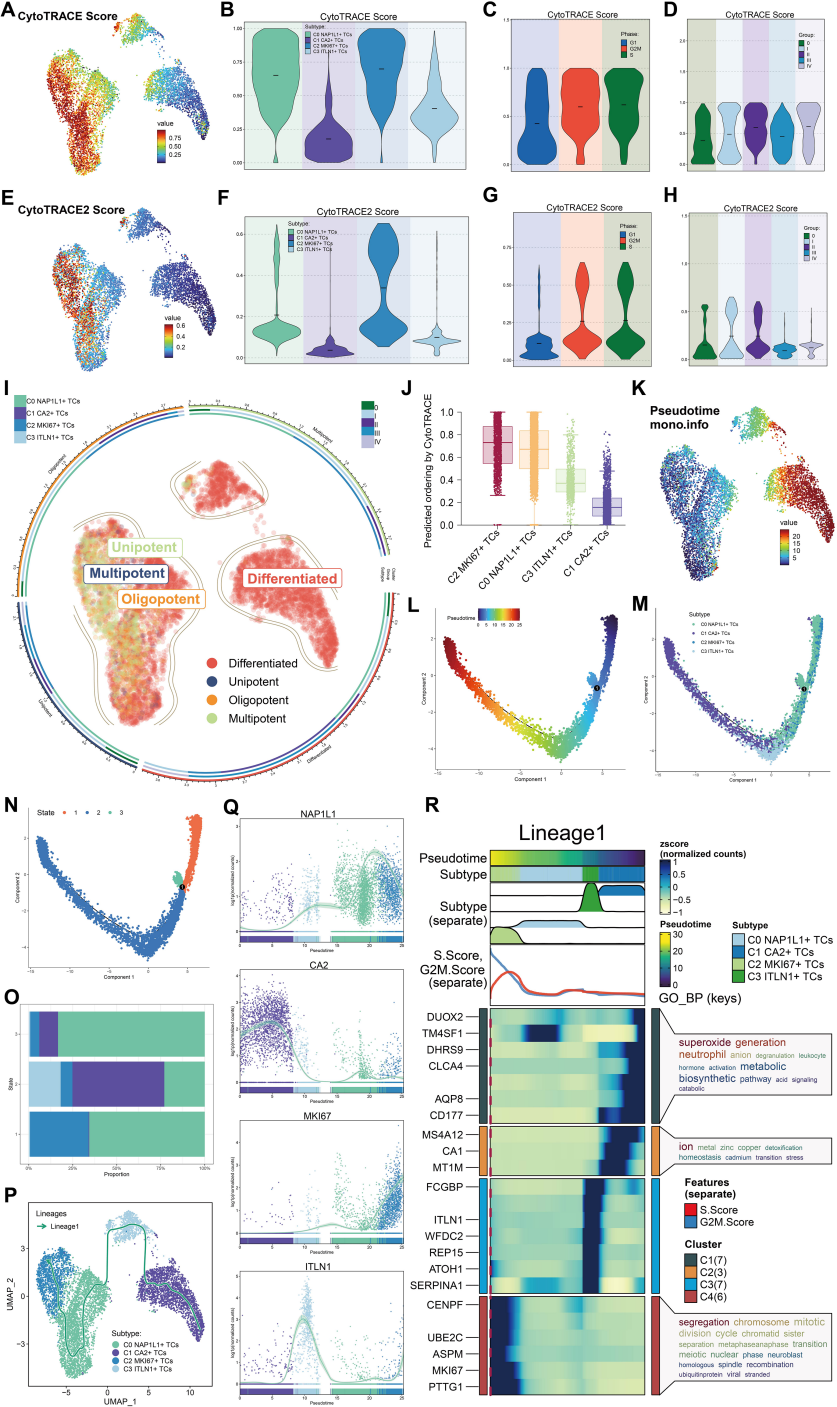
Potential for differentiation and developmental pathway of tumor cell subpopulations. **(A)** The UMAP visualization displayed CytoTRACE scores for all tumor cell subpopulations. **(B)** Violin plots illustrated the hierarchy of CytoTRACE scores among tumor cell subpopulations. **(C)** Variations in CytoTRACE scores were analyzed across distinct cell cycle phases. **(D)** Variations in CytoTRACE scores were analyzed among several tumor stage groups. **(E)** UMAP visualization displayed CytoTRACE2 scores across all tumor cell subpopulations. **(F)** Violin plots illustrated the hierarchy of CytoTRACE2 scores among tumor cell subpopulations. **(G)** Variations in CytoTRACE2 scores were analyzed throughout distinct cell cycle phases. **(H)** Variations in CytoTRACE2 scores were analyzed among several tumor stage groups. **(I)** Results of the CytoTRACE2_Potency study were presented for all tumor cell subpopulations. **(J)** Subpopulations of tumor cells were prioritized according to their differentiation capability, as shown by CytoTRACE scores. **(K)** Pseudotime ordering among all subpopulations was depicted using Monocle in UMAP space. **(L)** The developmental course of the pseudotime trajectory was depicted. **(M)** The distribution of all tumor cell subpopulations along the pseudotime trajectory was demonstrated. **(N)** The pseudotime trajectory was segmented into State 1 through State 3 according to branch points. **(O)** The proportions of each tumor cell subpopulation inside each pseudotime state were determined. **(P)** The developmental trajectory of Lineage 1 across tumor cell subpopulations was established using Slingshot. **(Q)** The expression dynamics of the naming genes *NAP1L1*, *CA2*, *MKI67*, and *ITLN1* along Lineage 1 were illustrated. **(R)** The GO-BP analysis was performed utilizing differentially expressed genes associated with Lineage 1.

We examined the differentiation capability of tumor cell subpopulations with CytoTRACE and CytoTRACE2. The UMAP visualization of CytoTRACE scores indicated that C2 *MKI67^+^* TCs and C0 *NAP1L1^+^* TCs exhibited comparatively elevated scores (**Figure 4A**). Violin plots further demonstrated that C2 *MKI67^+^* TCs exhibited the highest CytoTRACE score, succeeded by C0 *NAP1L1^+^* TCs (**Figure 4B**). Moreover, CytoTRACE scores were heightened in cells during the G2/M and S phases, reaching their zenith at tumor stage II, subsequently followed by stages IV, I, and III. Tumor stage 0 displayed the lowest CytoTRACE scores (**Figures 4C, D**). Correspondingly, UMAP visualization of CytoTRACE2 scores (**Figure 4E**) and violin plot analyses (**Figure 4F**) revealed that C2 *MKI67^+^* TCs exhibited the highest CytoTRACE2 score, succeeded by C0 *NAP1L1^+^* TCs. In accordance with CytoTRACE, CytoTRACE2 scores were elevated during the G2/M and S phases (**Figure 4G**). In contrast to CytoTRACE, CytoTRACE2 scores were elevated in tumor stages II and I, subsequently followed by stage 0 (**Figure 4H**). Moreover, the CytoTRACE2-Potency analysis characterized all tumor cell subpopulations, indicating that the majority of C1 *CA2^+^* TCs and C3 *ITLN1^+^* TCs were differentiated. Conversely, C2 *MKI67^+^* TCs and C0 *NAP1L1^+^* TCs—particularly C2 *MKI67^+^* TCs—encompassed cells at several differentiation stages, including differentiated, unipotent, oligopotent, and multipotent (**Figure 4I**). CytoTRACE findings together reveal that C2 *MKI67^+^* TCs possess the lowest differentiation and the highest differentiation potential, succeeded by C0 *NAP1L1^+^* TCs, whereas C1 *CA2^+^* TCs demonstrate the most differentiation and the lowest potential (**Figure 4J**). To further examine developmental disparities among tumor subpopulations, we utilized Monocle and Slingshot for trajectory inference. Monocle-based pseudotime ordering on UMAP elucidated the relative pseudotime positions of each subpopulation (**Figure 4K**). The established pseudotime trajectory progressed from right (early) to left (late) (**Figure 4L**). Mapping tumor subpopulations onto this trajectory revealed that C0 *NAP1L1^+^* TCs and C2 *MKI67^+^* TCs generally occupied the early pseudotime segment, C3 *ITLN1^+^* TCs were situated in the intermediate region, and C1 *CA2^+^* TCs were primarily found in the late segment (**Figure 4M**). At branching point 1, located at the commencement of the trajectory, we categorized the trajectory into three states: state 1–3 (**Figure 4N**). State1 and state3 denoted the initial pseudotime, whereas state2 encompassed the latter portion of the early segment, the midpoint, and the late pseudotime. State 1 was characterized by a predominance of C0 *NAP1L1^+^* TCs and C2 *MKI67^+^* TCs; state 2 exhibited an enrichment of C1 *CA2^+^* TCs, succeeded by C0 *NAP1L1^+^* TCs and C3 *ITLN1^+^* TCs; state 3 was mostly constituted of C0 *NAP1L1^+^* TCs (**Figure 4O**). Subsequently, we utilized Slingshot to reconstruct a lineage trajectory, designated Lineage1, with the inferred sequence: C1 *CA2^+^* TCs → C3 *ITLN1^+^* TCs → C0 *NAP1L1^+^* TCs → C2 *MKI67^+^* TCs (**Figure 4P**). The expression trends of hallmark genes along Lineage1 indicated that *NAP1L1* and *MKI67* elevated towards the trajectory’s conclusion, *CA2* was predominant at the onset, and *ITLN1* reached its zenith in the intermediate segment (**Figure 4Q**). Differential expression study across Lineage1 stages indicated that at the terminal stage characterized by C2 *MKI67^+^* TCs, genes including *CENPF*, *UBE2C*, *ASPM*, *MKI67*, and *PTTG1* exhibited elevated expression levels. The GO-BP enrichment analysis of these genes underscored their participation in processes such as chromosome segregation, mitotic division, cell cycle regulation, and chromatid organization (**Figure 4R**).

### Enrichment analysis of tumor cell subpopulations in CRC

**Figure 5:**
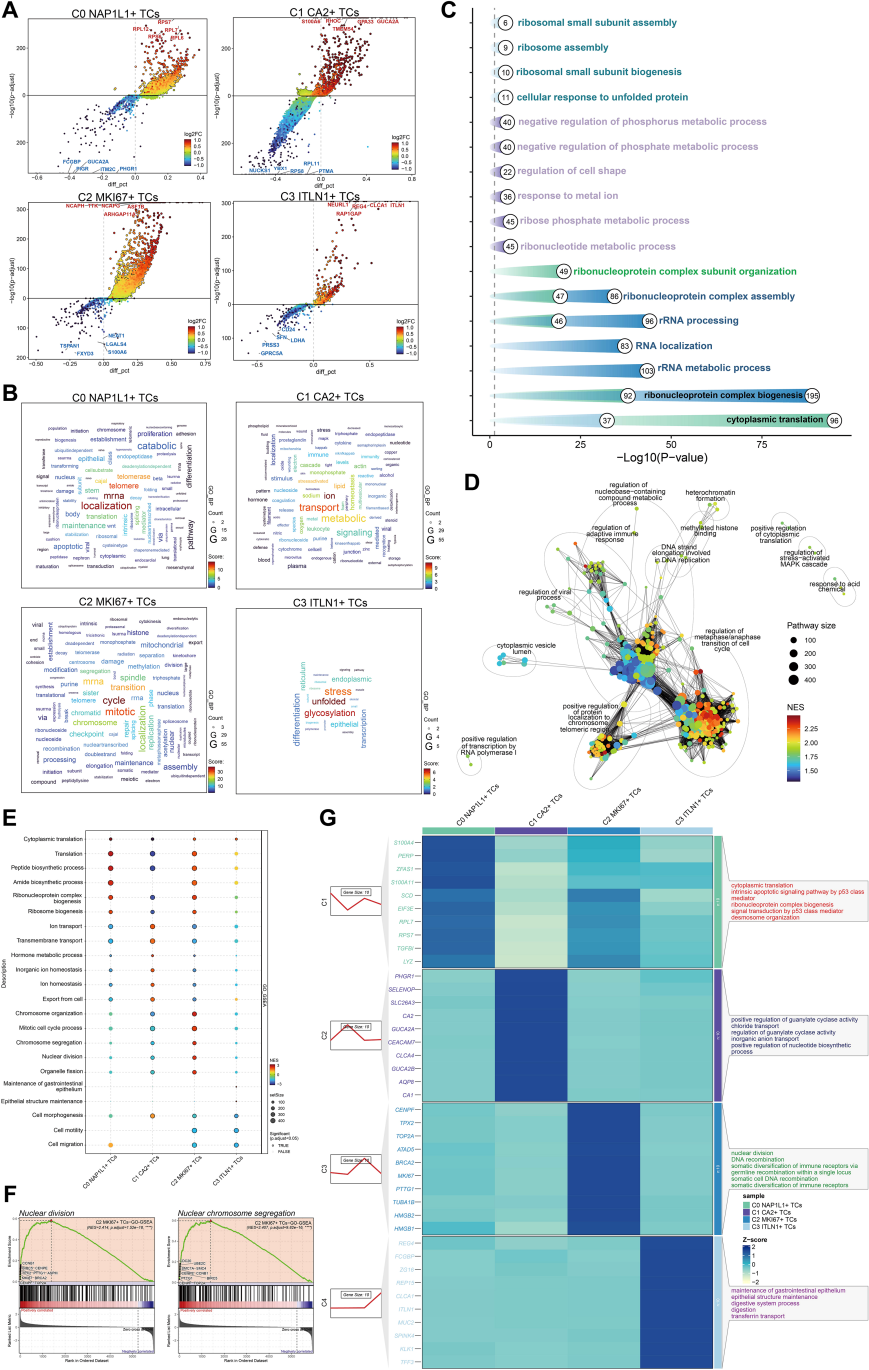
Analysis of enrichment in tumor cell subpopulations. **(A)** Volcano plots illustrated the five most upregulated and five most downregulated genes within each of the four tumor cell subpopulations. **(B)** Word clouds depicted the primary enriched phrases linked to each tumor cell subpopulations. **(C)** GO enrichment analysis were conducted for the four tumor cell subpopulations. **(D)** A network map of enrichment was created for pathways related to C2 *MKI67^+^* TCs. **(E)** GO-GSEA analysis were performed for the four tumor cell subpopulations. **(F)** Two representative GO-GSEA enrichment keywords associated to C2 *MKI67^+^* TCs were highlighted. **(G)** GO-BP analysis was conducted utilizing the top 10 differentially expressed genes from each tumor cell subpopulation.

We employed volcano plots to ascertain the five most upregulated and five most downregulated genes for each tumor cell subpopulation (**Figure 5A**). In C0 *NAP1L1^+^* TCs, the five most upregulated genes were *RPL12*, *RPS6*, *RPS7*, *RPL7*, and *RPL6*, whereas the five most downregulated genes were *FCGBP*, *PIGR*, *GUCA2A*, *ITM2C*, and *PHGR1*. In C1 *CA2^+^* TCs, the five most upregulated genes were *S100A6*, *RHOC*, *TMEM54*, *GPA33*, and *GUCA2A*, while the five most downregulated genes were *NUCKS1*, *YBX1*, *RPS8*, *RPL11*, and *PTMA*. In C2 *MKI67^+^* TCs, the five most upregulated genes were *NCAPH*, *TTK*, *NCAPG*, *ASF1B*, and *ARHGAP11A*, whereas the five most downregulated genes were *TSPAN1*, *FXYD3*, *NEAT1*, *LGALS4*, and *S100A6*. In C3 *ITLN1^+^* TCs, the five most upregulated genes were *NEURL1*, *RAP1GAP*, *REG4*, *CLCA1*, and *ITLN1*, while the five most downregulated genes were *CD24*, *SFN*, *LDHA*, *PRSS3*, and *GPRC5A*. Word cloud analysis indicated that C0 *NAP1L1^+^* TCs were significantly associated with terms including localization, mRNA, telomere, and telomerase; C1 *CA2^+^* TCs correlated with terms such as ion, transport, lipid, stress-activated, and metabolic; C2 *MKI67^+^* TCs were connected to cycle, mitotic, and transition-related terms; and C3 *ITLN1^+^* TCs were associated with unfolded protein response, glycosylation, and stress (**Figure 5B**). GO enrichment analysis of DEGs within each subpopulation indicated that C2 *MKI67^+^* TCs were predominantly linked to ribonucleoprotein complex formation, rRNA metabolic processes, RNA localization, rRNA processing, and ribonucleoprotein complex assembly (**Figure 5C**). The enrichment network for C2 *MKI67^+^* TCs emphasized pathways including the regulation of the metaphase/anaphase transition in the cell cycle, the positive regulation of protein localization to chromosomes and telomeric regions, and the regulation of the adaptive immune response (**Figure 5D**). GO-GSEA analysis revealed that C2 *MKI67^+^* TCs were significantly linked to chromosome organization, mitotic cell cycle processes, chromosome segregation, nuclear division, organelle fission, and ribonucleoprotein complex biogenesis (**Figure 5E**). Two sample enriched phrases, nuclear division and nuclear chromosome segregation, were illustrated in detail (**Figure 5F**).

A heatmap illustrated the DEGs across subpopulations, revealing that C2 *MKI67^+^* TCs exhibited elevated expression levels of *CENPF*, *TPX2*, *TOP2A*, *ATAD5*, *BRCA2*, *MKI67*, *PTTG1*, *TUBA1B*, *HMGB2*, and *HMGB1*. GO-BP analysis of these genes demonstrated significant enrichment in nuclear division, DNA recombination, somatic diversification of immune receptors through germline recombination at a single locus, somatic cell DNA recombination, and somatic diversification of immune receptors (**Figure 5G**).

### C2 *MKI67^+^* TCs interact with immune-related cells through the MIF-(CD74+CD44) axis

**Figure 6:**
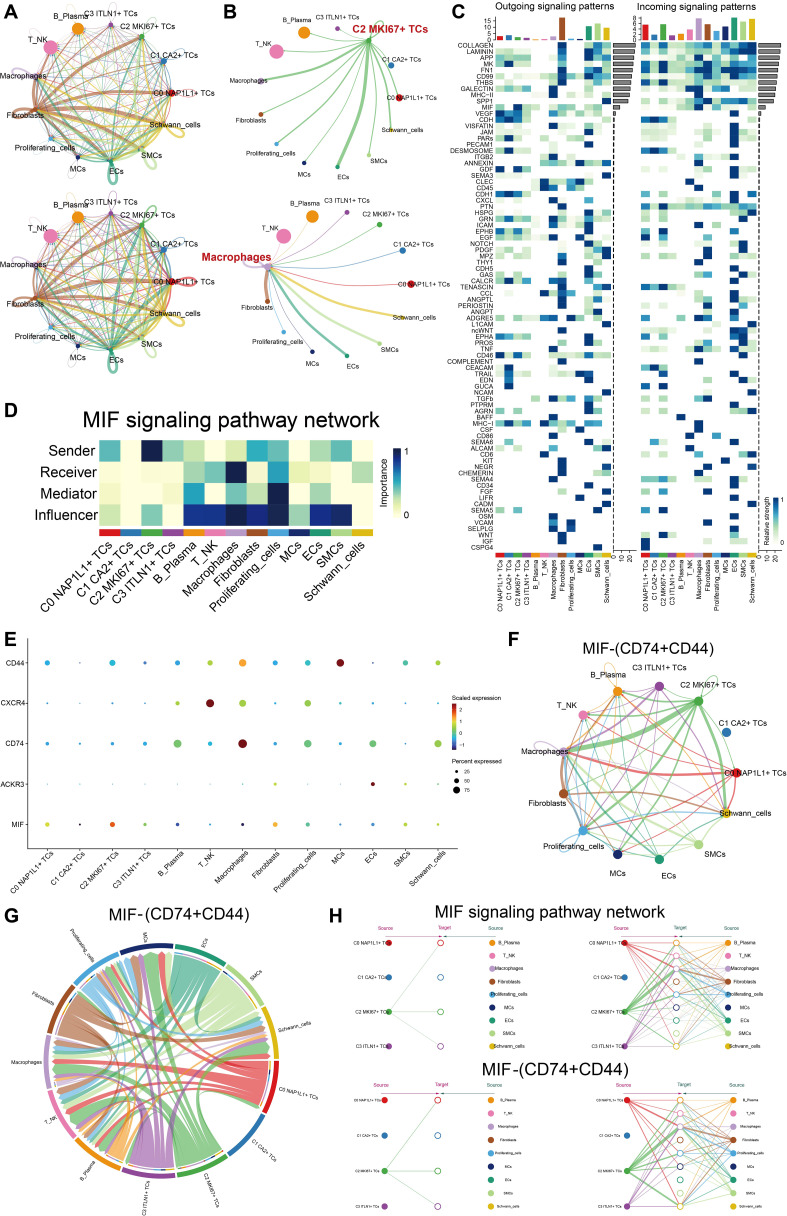
C2 *MKI67^+^* TCs engaged with immune cells through the MIF-(CD74+CD44) signaling pathway. **(A)** The circos plots depicted the interaction intensity and quantity among all tumor cell subpopulations and all cell types. **(B)** The upper panel illustrated the contacts initiated by C2 *MKI67^+^* T cells with other cell types, whereas the lower panel represented the interactions directed toward macrophages by various cell types. **(C)** The expression levels of ligand-receptor pairings were shown with all cells functioning as either signal senders or receivers. **(D)** The functions of all cell types as transmitters, receptors, mediators, and modulators were evaluated in *MIF*-related signaling pathways. **(E)** Receptor expression in other cells was shown when *MIF*, highly expressed in C2 *MKI67^+^* TCs, acted as the ligand. F–G. Circos and chord plots visualized the intercellular communication through the MIF-(CD74+CD44) signaling pathway. **(H)** A hierarchical communication map depicted cell-cell signaling facilitated by *MIF*-related and MIF-(CD74+CD44) signaling pathways, with the upper and bottom layers denoting sender and recipient cell types, respectively; arrows showed the direction of signal flow, and line thickness denoted the strength of communication.

We depicted intercellular communication across all cell types with CellChat. A chord diagram illustrated the total intensity and quantity of interactions among all cells (**Figure 6A**). In the chord diagram illustrating the signaling from the important subpopulation C2 *MKI67^+^* TCs to other cells, the interaction intensity from C2 *MKI67^+^* TCs to macrophages and ECs was significantly greater. Concurrently, macrophages acquired enhanced signaling from fibroblasts, ECs, SMCs, and Schwann cells (**Figure 6B**). The heatmap illustrating receptor-ligand expression patterns across all cells is presented for both outgoing and incoming signaling patterns (**Figure 6C**). MIF was significantly expressed in C2 *MKI67^+^* TCs within the outgoing signaling pathways. Consequently, we concentrated on the *MIF*-associated signaling pathway for enhanced visualization. Within the *MIF* signaling network, C2 *MKI67^+^* TCs demonstrated a significant sender importance score, while macrophages displayed a notably high receiver important score (**Figure 6D**). Subsequent comprehensive investigation indicated that *MIF* was significantly expressed in C2 *MKI67^+^* TCs, *CD74* was mostly found in macrophages, and *CD44* was detected in both MCs and macrophages (**Figure 6E**). Through chord diagrams and circular plots, we illustrated that the communication strength along the MIF-(CD74+CD44) axis was much greater between C2 *MKI67^+^* TCs and macrophages (**Figures 6F, G**). A hierarchy diagram ultimately validated that across both the *MIF* signaling network and the MIF-(CD74+CD44) axis, C2 *MKI67^+^* TCs exert a significant paracrine influence on macrophages (**Figure 6H**).

### Transcriptional regulatory characteristics of principal tumor subpopulations

**Figure 7:**
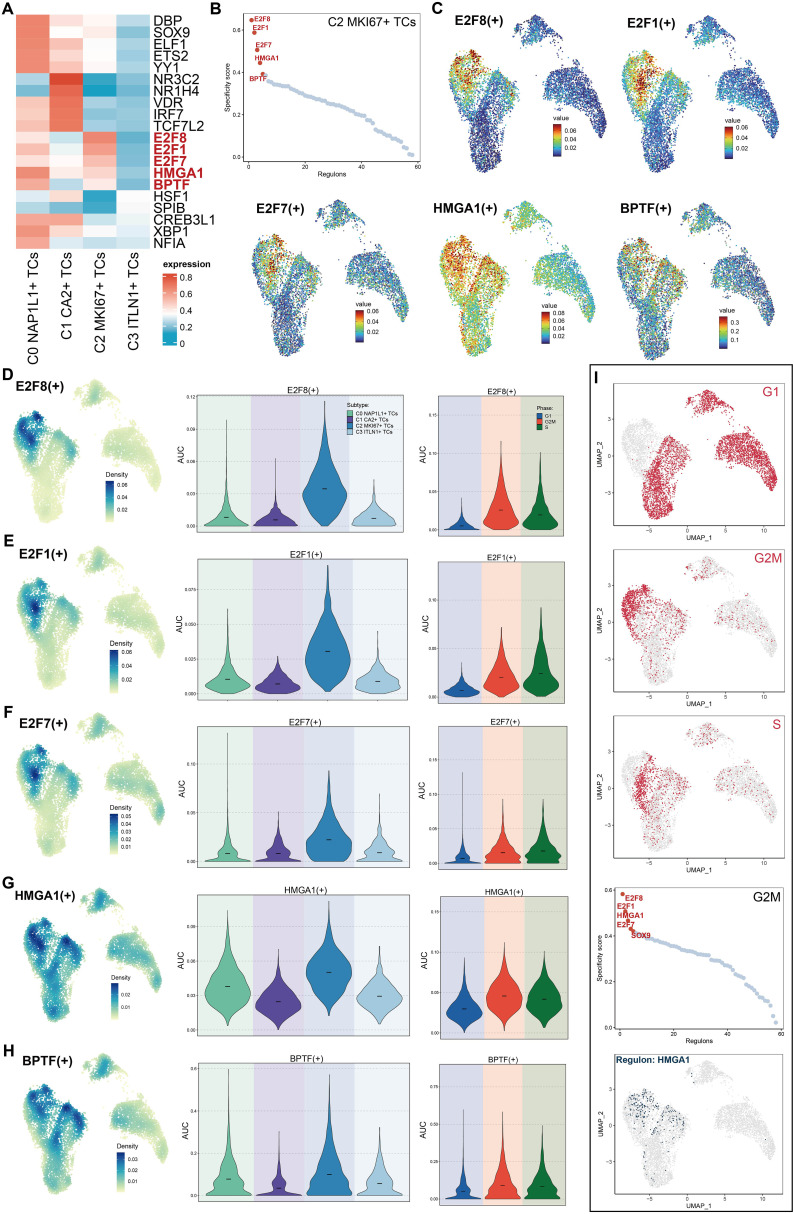
Characteristics of TFs activity in C2 *MKI67^+^* T cells. **(A)** The heatmap displayed the five most active TFs among the four tumor cell subpopulations. **(B)** The five foremost TFs in C2 *MKI67^+^* TCs, sorted by specificity score, were provided. **(C)** UMAP plots illustrated the five most active TFs in C2 *MKI67^+^* TCs. **(D–H)**. The relative densities of the five most active TFs in C2 *MKI67* ^+^ TCs were proved. The Violin graph displayed the AUC values of these five TFs in subpopulations and their changes at the cell cycle stage. **(I)** UMAP plots initially illustrated the distribution of cells across G1, G2/M, and S phases within subpopulations (top). In cells during the G2/M phase, the five TFs with the highest specificity scores were enumerated (center). The expression distribution of *HMGA1* among subpopulations was depicted using UMAP (bottom).

We depicted the five principal transcription factors (TFs) across all tumor subpopulations utilizing a heatmap (**Figure 7A**). The five most prominent transcription factors in the C2 *MKI67^+^* T cell subpopulation were *E2F8*, *E2F1*, *E2F7*, *HMGA1*, and *BPTF*. Furthermore, we evaluated all TFs in C2 *MKI67^+^* TCs according to their specificity scores, producing results that align with those presented in the heatmap (**Figure 7B**). Subsequently, we depicted the expression of these five predominant TFs on a UMAP plot, revealing elevated expression levels solely within the C2 *MKI67^+^* T cell subpopulation (**Figure 7C**). The relative expression levels of these five TFs were elevated in C2 *MKI67^+^* tumor cells (**Figures 7D-H**). Additionally, violin plots were employed to effectively compare the expression levels of the five TFs across various tumor cell subpopulations and cell cycle phases. All five TFs exhibited peak expression in C2 *MKI67^+^* tumor cells. *HMGA1* expression was most elevated in C2 *MKI67^+^* TCs, followed by a comparatively high expression in C0 *NAP1L1^+^* TCs. *E2F8*, *HMGA1*, and *BPTF* exhibited increased expression in cells during the G2/M phase; *E2F1* was primarily expressed in the S phase; and *E2F7* demonstrated no substantial difference between the G2/M and S phases, although it was marginally elevated in the S phase. Subsequently, cells in the G1, G2/M, and S phases were distinctly seen on UMAP plots (**Figure 7I**), corroborating prior results that cells in the G2/M phase predominantly clustered within the C2 *MKI67^+^* TCs group. Concentrating on G2/M phase cells, we ranked all TFs by their specificity scores, identifying *E2F8*, *E2F1*, *HMGA1*, *E2F7*, and *SOX9* as the five most prominent. *HMGA1* was distinctly represented on a UMAP plot, exhibiting significant expression in C2 *MKI67^+^* TCs.

### Transcriptional regulatory variability of tumor cell subpopulations

**Figure 8:**
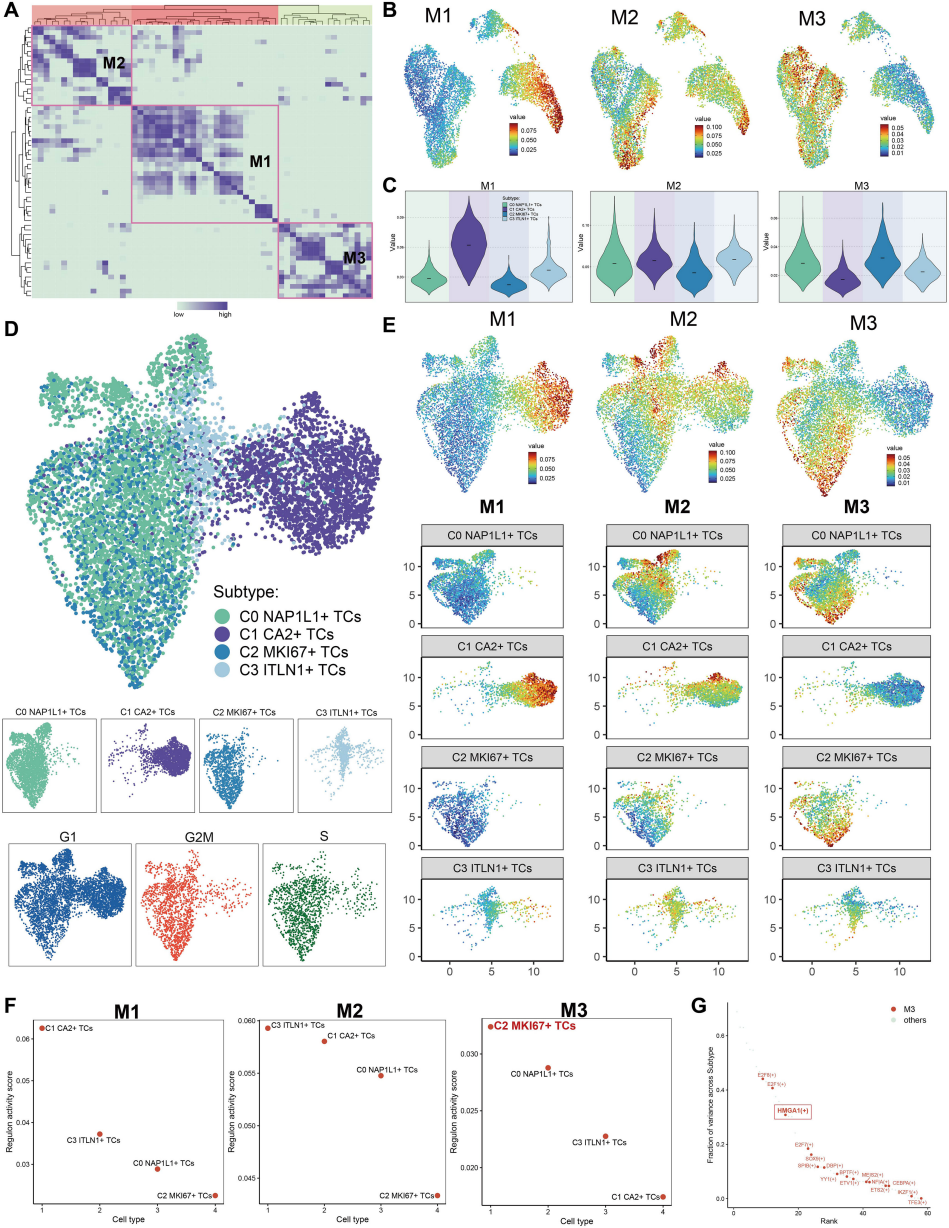
The construction of regulatory modules defined the transcriptional regulatory heterogeneity of tumor cell subpopulations. **(A)** The TFs of tumor cell subpopulations were categorized into three regulatory modules (M1, M2, M3) according to the CSI matrix. **(B)** The primary distribution of the three regulatory modules on the original UMAP plot was displayed. **(C)** The Violin images were used to show the proportion ranking of tumor subpopulations in the three regulatory modules. **(D)** The UMAP plot was generated through re-clustering utilizing pySCENIC analysis and TFs regulatory activity in CRC tumor cells. The UMAP illustrated the distribution of subpopulations, while facet plots depicted the distribution of cells in the G1, G2/M, and S phases. **(E)** The allocation of the three regulatory modules and the tumor cell subpopulations across various modules was illustrated in the new UMAP figure. **(F)** The rankings of regulon activity ratings for tumor subpopulations across the three regulatory modules were reported. **(G)** In regulatory module M3, TFs were prioritized according to the variation fraction across subtypes and presented.

Utilizing the CSI matrix, we categorized the TFs of all tumor cell subpopulations into three regulatory modules, labeled M1, M2, and M3 (**Figure 8A**). Subsequently, we illustrated the distribution of M1, M2, and M3 throughout all subpopulations utilizing UMAP plots (**Figure 8B**). M1 was mostly concentrated in C1 *CA2^+^* TCs; M2 was chiefly dispersed throughout C0 *NAP1L1^+^* TCs, C1 *CA2^+^* TCs, and C3 *ITLN1^+^* TCs; whereas M3 was predominantly linked to C2 *MKI67^+^* TCs. Violin plots effectively illustrated the expression levels of these regulatory modules among tumor subpopulations, with C2 *MKI67^+^* TCs exhibiting the highest expression in M3, succeeded by C0 *NAP1L1^+^* TCs (**Figure 8C**). Furthermore, we executed pySCENIC analysis and implemented a novel dimensionality reduction and clustering based on the regulatory activity of TFs in CRC tumor cells, culminating in a new UMAP visualization depicted in **Figure 8D**. Facet plots illustrated the distributions of tumor subpopulations and cells in the G1, G2/M, and S phases. M1, M2, and M3 were subsequently displayed on the new UMAP plot (**Figure 8E**). The findings revealed that C1 *CA2^+^* TCs constituted a greater percentage in M1; C0 *NAP1L1^+^* TCs and C1 *CA2^+^* TCs were more prevalent in M2; and C2 *MKI67^+^* TCs and C0 *NAP1L1^+^* TCs were predominant in M3. These results aligned with the earlier visual representations derived from the original UMAP graphic. Subpopulations of tumors within M1, M2, and M3 were subsequently graded based on their regulon activity scores (**Figure 8F**). In M1, C1 *CA2^+^* TCs were ranked highest, whereas C2 *MKI67^+^* TCs were ranked lowest. In M2, C3 *ITLN1^+^* TCs achieved the highest ranking, while C2 *MKI67^+^* TCs rated the lowest once more. In M3, C2 *MKI67^+^* TCs were the most prominent. Ultimately, we prioritized TFs inside M3 according to the proportion of variance between subtypes, highlighting *E2F8*, *E2F1*, and *HMGA1* as the foremost candidates (**Figure 8G**).

### Differential evaluation of heart failure-associated genes among CRC tumor cell subpopulations

**Figure 9:**
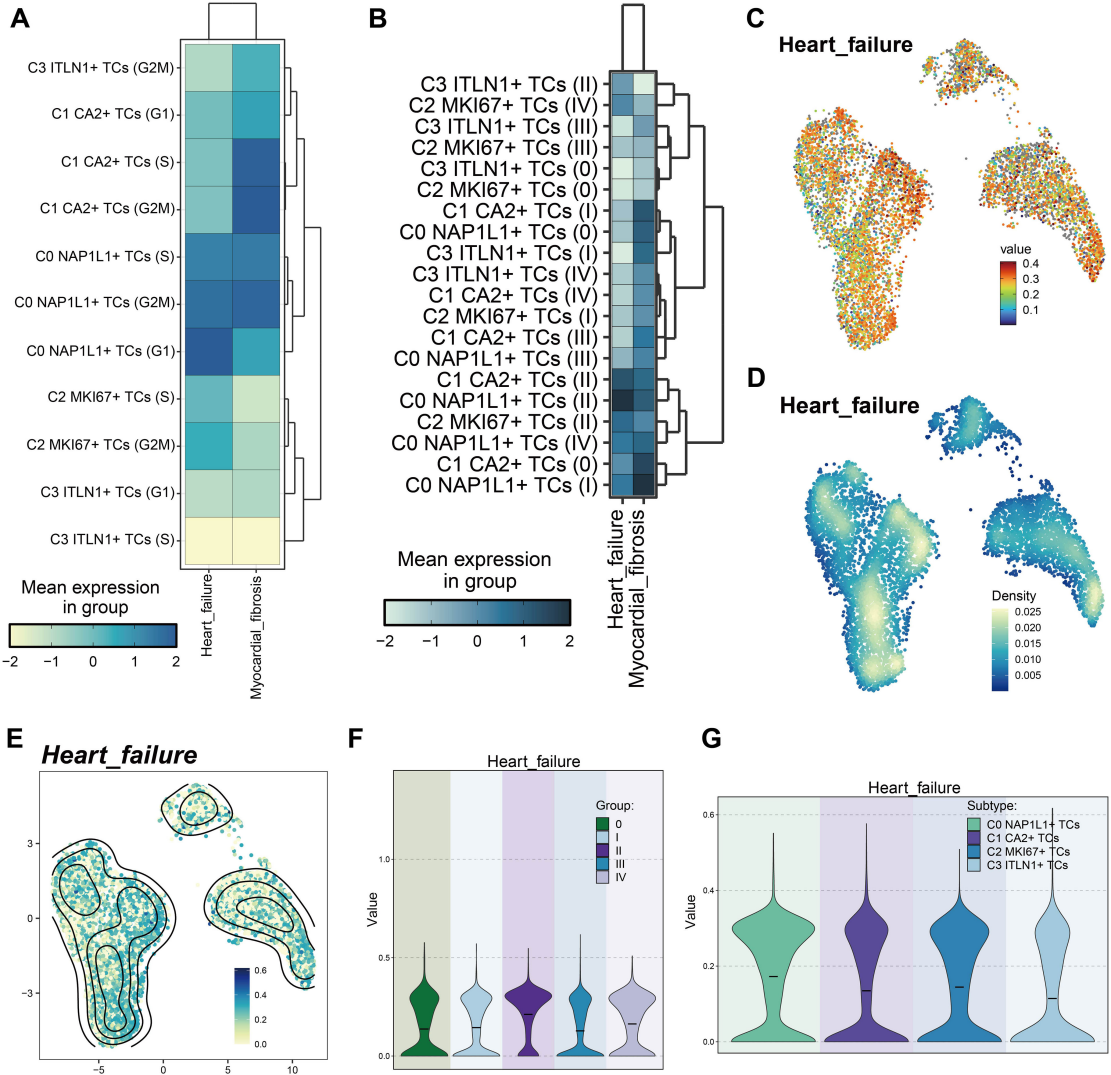
Variations in heart failure-associated gene set scores among tumor cell subpopulations. **(A)** Heatmap demonstrated the heart failure and cardiac fibrosis scores of tumor cell subpopulations at distinct cell cycle stages. **(B)** The heatmap displayed the heart failure and cardiac fibrosis scores of tumor cell subpopulations across various tumor stages. **(C)** UMAP illustrated the heart failure scores among tumor subpopulations. **(D, E)**. Comparisons of relative density of heart failure scores among tumor cell subpopulations were presented. **(F)** The violin plot intuitively compares the ranks of heart failure scores across various tumor stages. **(G)** The violin plot intuitively contrasted the rankings of heart failure scores among tumor subpopulations.

To investigate the possible connections between CRC tumor cell subpopulations and cardiac dysfunction, which may result in heart failure, we assembled a gene collection associated with heart failure and myocardial fibrosis. This gene set was compiled from current research and established consensus, encompassing genes expressed in colorectal tumor tissues that are recognized to be linked to heart failure and myocardial fibrosis. Utilizing this gene set, we evaluated each tumor cell subpopulation for its association with heart failure and myocardial fibrosis. Initially, we categorized tumor subpopulations based on cell cycle stages (**Figure 9A**). Heart failure scores were elevated in C0 *NAP1L1^+^* TCs (G1), C0 *NAP1L1^+^* TCs (G2/M), C0 *NAP1L1^+^* TCs (S), C2 *MKI67^+^* TCs (G2/M), and C2 *MKI67^+^* TCs (S). Concurrently, cardiac fibrosis scores were heightened in C0 *NAP1L1^+^* TCs (G2/M), C0 *NAP1L1^+^* TCs (S), C1 *CA2^+^* TCs (G2/M), and C1 *CA2^+^* TCs (S). Subsequently, we evaluated tumor subpopulations across various tumor stages (**Figure 9B**). The cardiac failure scores were significantly elevated in C0 *NAP1L1^+^* TCs (stage II), C1 *CA2^+^* TCs (stage II), and C2 *MKI67^+^* TCs (stage II). Myocardial fibrosis scores were heightened in C0 *NAP1L1^+^* TCs (stage I), C1 *CA2^+^* TCs (stage 0), C1 *CA2^+^* TCs (stage I), and C0 *NAP1L1^+^* TCs (stage II). We additionally illustrated the expression pattern and density of heart failure scores among subpopulations by UMAP plots (**Figures 9C–E**), indicating that peak values are primarily concentrated in C0 *NAP1L1^+^* TCs and C2 *MKI67^+^* TCs. Violin plots comparing heart failure ratings throughout tumor stages indicated that stage II tumors had the highest scores, succeeded by stage IV (**Figure 9F**). Violin plots illustrating inter-subpopulation disparities indicated that C0 *NAP1L1^+^* TCs and C2 *MKI67^+^* TCs possessed considerably elevated heart failure scores, whereas C1 *CA2^+^* TCs demonstrated moderate values, and C3 *ITLN1^+^* TCs displayed the lowest scores (**Figure 9G**).

### *In vitro* functional validation of *HMGA1*

**Figure 10:**
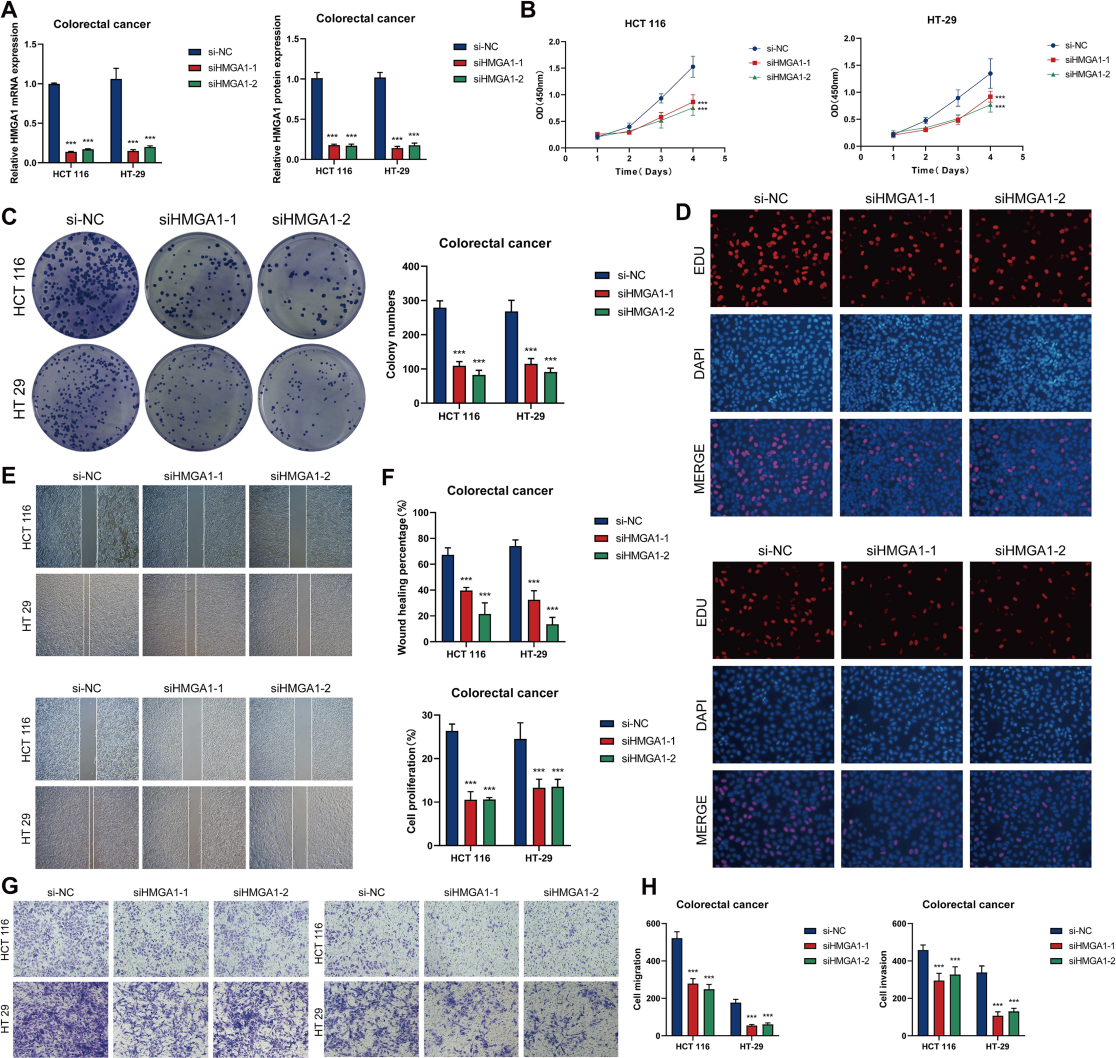
*In vitro* investigations of *HMGA1* suppression. **(A)** The mRNA and protein expression levels of *HMGA1* in the HCT116 and HT-29 cell lines were assessed across three groups. si-NC, si*HMGA1*-1, and si*HMGA*1-2. *HMGA1* knockdown markedly reduced both mRNA and protein expression levels. **(B)** CCK-8 assays indicated a substantial reduction in cell viability following *HMGA1* knockdown in both HCT116 and HT-29 cell lines. **(C)** Colony formation assays revealed a substantial reduction in the number of colonies in HCT116 and HT-29 cells subsequent to *HMGA1* knockdown. **(D)** EDU labeling revealed that *HMGA1* knockdown suppressed cell growth. **(E)** Wound healing tests demonstrated that *HMGA1* knockdown inhibited cell migration. **(F)** The rates of wound healing and cell proliferation were markedly diminished following *HMGA1* knockdown. **(G, H)** Transwell tests demonstrated that *HMGA1* knockdown impeded cell migration and invasion in HCT116 and HT-29 cells. ***P < 0.001.

We conducted pertinent *in vitro* research centered on the *HMGA1* gene to investigate its function as a principal transcription factor in C2 *MKI67^+^* tumor cells. *HMGA1* knockdown was performed on two CRC cell lines, HCT116 and HT-29. After knockdown, the mRNA and protein expression levels of *HMGA1* were markedly diminished in both cell lines (**Figure 10A**). Moreover, in comparison to control groups, cell viability was significantly reduced in both cell lines following *HMGA1* knockdown (**Figure 10B**). Colony formation experiments revealed a marked decrease in colony numbers following *HMGA1* silencing (**Figure 10C**). The EDU labeling experiments demonstrated that decreased *HMGA1* levels impeded cell growth (**Figure 10D**). Furthermore, wound healing studies demonstrated that *HMGA1* knockdown not only inhibited cell migration (**Figure 10E**) but also markedly reduced the wound closure rate and cellular proliferation capability (**Figure 10F**). Transwell experiments demonstrated that the migratory and invasive capacities of HCT116 and HT-29 cells were significantly diminished after *HMGA1* knockdown (**Figures 10G, H**).

### Construction of the risk scoring prediction model

**Figure 11:**
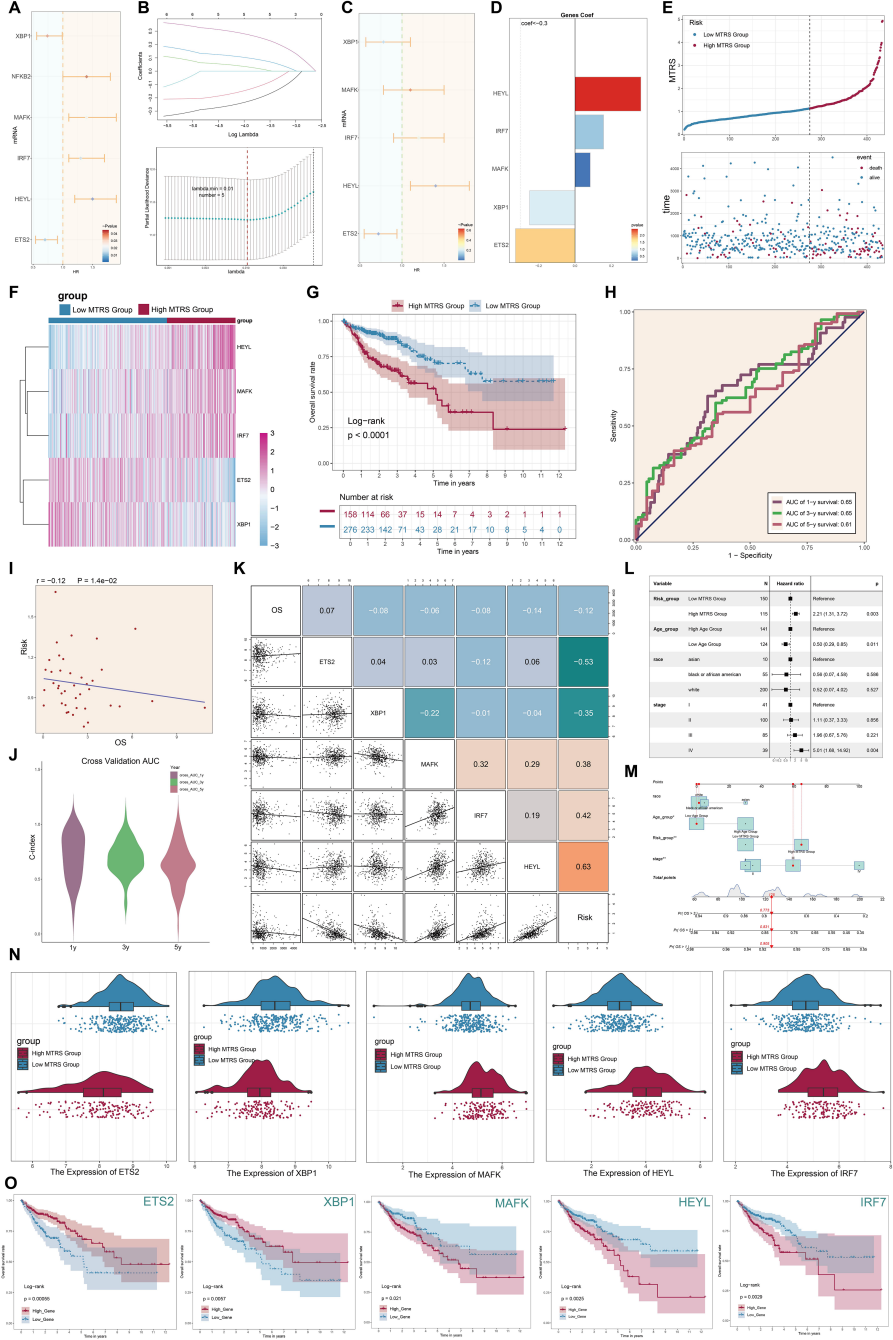
Development of the *MKI67^+^* TCs risk score (MTRS) model. **(A)** The forest plot displayed genes with substantial disparities in univariate Cox regression analysis (HR < 1 denoted protective variables; HR > 1 denoted risk factors). **(B)** LASSO regression analysis discovered five prognosis-associated genes. Each line indicated a gene with a distinct coefficient related to prognosis, and the optimal parameter was determined by cross-validation (top). The LASSO coefficient curve was constructed based on the optimal lambda value (bottom). **(C)** The forest plot illustrated the five prognosis-associated genes found using multivariate Cox regression analysis. **(D)** The coefficient (coef) values of the genes utilized to develop the risk score model were presented. **(E)** The line graph displayed the disparities in risk scores between the high and low MTRS groups, while the scatter plot illustrated survival and mortality events across time in both groups. **(F)** The heatmap illustrated the differential expression of the five risk genes between the high and low MTRS groups. **(G)** The Kaplan–Meier survival curves illustrated the disparity in survival rates over time between the high and low MTRS groups. **(H)** The AUC for forecasting 1-year, 3-year, and 5-year outcomes in the cohort was demonstrated. **(I)** The scatter plot illustrated the correlation between risk scores an OS. **(J)** The violin plot clearly contrasted the differences in the C-index at 1, 3, and 5 years during cross-validation. **(K)** The relationships among prognosis-related genes, OS, and the genes utilized to develop the model were demonstrated. **(L)** The forest plot displayed a multivariate Cox regression analysis that incorporated risk scores and clinical variables such as age, race, and tumor stage. **(M)** The nomogram forecasted 1-, 3-, and 5-year overall survival based on risk scores, age, ethnicity, and clinical tumor stages (I, II, III, IV). **(N)**. The differential expression of prognosis-related genes between the high and low MTRS groups was demonstrated. **(O)**. Kaplan–Meier survival curves analyzed survival disparities between high-expression and low-expression cohorts for each of the five risk-associated genes. *P<0.05, **P<0.01.

To study the potential clinical relevance of the *MKI67^+^/HMGA1* regulatory network, we created a risk prediction model based on the major tumor cell subpopulation C2 *MKI67^+^* TCs. First, univariate Cox regression analysis was done to identify significantly differentially expressed genes and evaluate their prognostic significance (**Figure 11A**). Subsequently, LASSO regression analysis was employed to identify genes significantly associated with prognosis (**Figure 11B**). Multivariate Cox regression analysis found *MAFK*, *IRF7*, and *HEYL* as independent negative prognostic variables (hazard ratio [HR] > 1) (**Figure 11C**). Subsequently, we computed the coefficient values of the chosen genes to assess their correlation with survival outcomes (**Figure 11D**). Utilizing the relevant formula, we calculated an *MKI67^+^* TCs risk score (MTRS) for each patient and conducted a differential gene expression study. Patients in the TCGA cohort were categorized into two groups, High MTRS and Low MTRS, utilizing the best cutoff point for MTRS. Survival analysis through scatter plots indicated that the High MTRS group demonstrated markedly reduced survival rates and a worse prognosis (**Figure 11E**). A heatmap depicted the differential expression of five model-related genes across the two groups, revealing increased expression of *HEYL*, *MAFK*, and *IRF7* in the High MTRS group, and heightened expression of *ETS2* and *XBP1* in the Low MTRS group, corroborating prior findings (**Figure 11F**). The Kaplan-Meier survival curves showed that the High MTRS group had significantly worse survival outcomes compared to the Low MTRS group (**Figure 11G**). ROC curves demonstrated the model’s predictive accuracy and consistency, exhibiting AUC values at 1-, 3-, and 5-year intervals within the TCGA cohort (**Figure 11H**). Furthermore, risk ratings exhibited a negative correlation with OS, suggesting that elevated risk scores forecasted diminished OS (**Figure 11I**). The model’s accuracy was further validated by calculating the C-index, which resulted in values exceeding 0.5 at 1, 3, and 5 years, confirming its strong predictive ability (**Figure 11J**). Correlation heatmaps and scatter plots were created to thoroughly analyze the correlations among the five model genes, risk scores, and OS (**Figure 11K**). Additionally, subpopulation analyses based on race, age, tumor stages (I, II, III, IV), and risk scores were performed to assess their varying effects on OS (**Figures 11L, M**). In the end, **Figure 11**F’s heatmap provided a detailed visualization of the expression differences of the five model genes between the two risk groups (**Figure 11N**). Kaplan-Meier survival analyses revealed significant disparities in survival between groups exhibiting high and low expression of these genes (**Figure 11O**). Elevated expression of *ETS2* and *XBP1* connected with improved survival, aligning with their protective functions, while increased expression of *MAFK*, *HEYL*, and *IRF7* was linked to lower survival outcomes, reinforcing their status as negative prognostic indicators.

### Analysis of immune infiltration and drug sensitivity in high- and low-risk groups

**Figure 12:**
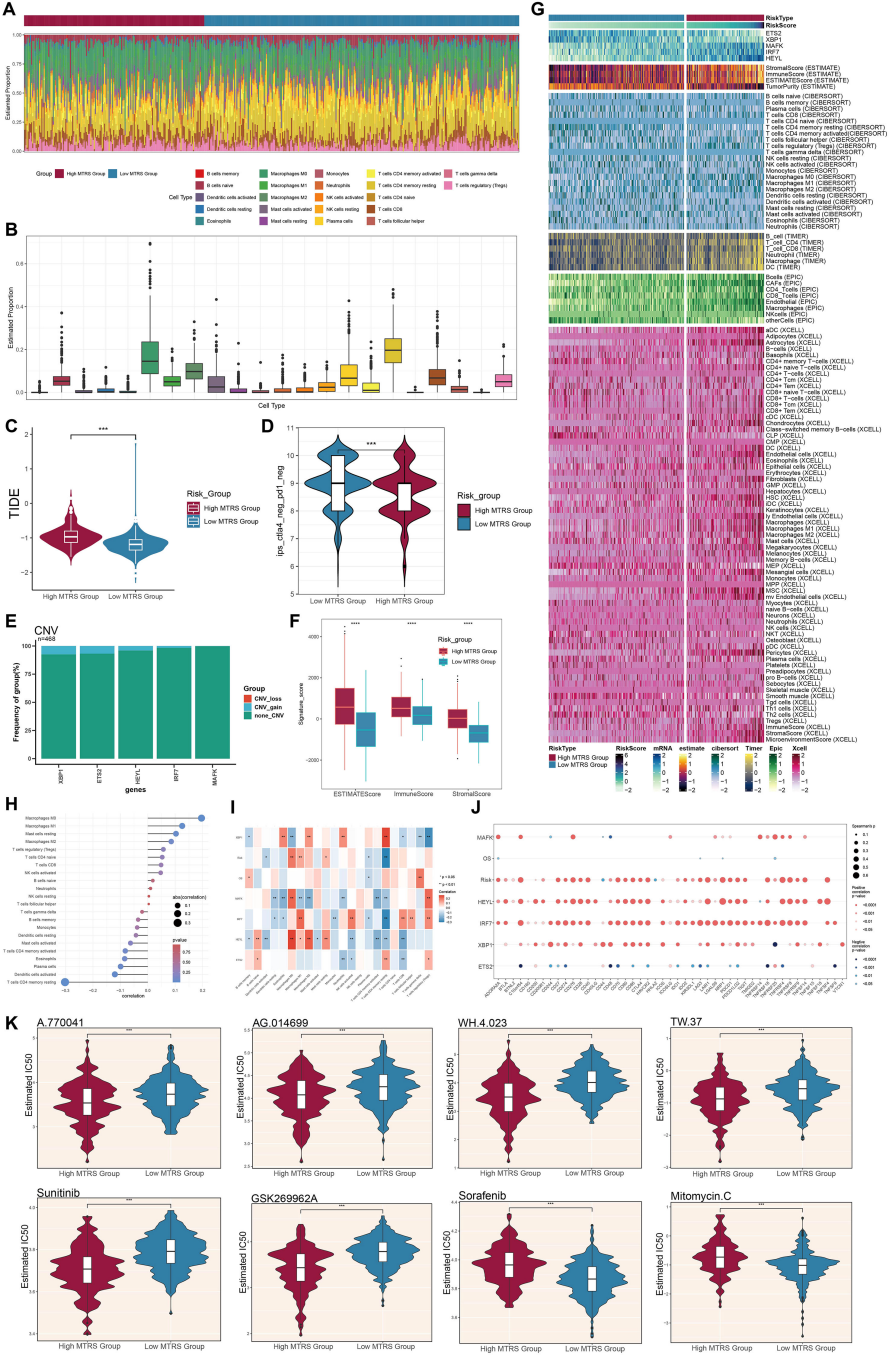
Variations in immune infiltration and pharmacological sensitivity between elevated and diminished MTRS cohorts. **(A, B)** The ratios of 22 immune cell types were assessed and contrasted between the high and low MTRS groups. **(C)** Violin plots illustrated the disparities in TIDE expression levels between the two risk categories. **(D)** Violin plots illustrated variations in immunological phenotype scores (IPS) between the two risk categories. **(E)** Bar plots depicted the amplification or deletion of CNVs for five genes pertinent to the creation of the risk model. **(F)** The disparities in stromal score, immune score, and ESTIMATE score between the high and low MTRS groups were examined. **(G)** The heatmap illustrated the distinct variations in gene expression between the two groups regarding the model, stromal score, immunological score, ESTIMATE score, tumor purity, and levels of immune cell infiltration, as determined by the CIBERSORT and Xcell algorithms. **(H, I)** Correlation analyses were conducted among the five model-associated genes, risk scores, OS, and immune-related cells. **(J)** Bubble plots depicted the correlations among model genes, risk scores, OS, and immune checkpoint-related genes. **(K)** Violin graphs examined the IC50 values of various chemotherapeutic agents between the high and low MTRS groups. *P < 0.05, **P < 0.01, ***P < 0.001, ****P < 0.0001; “ns” indicated no significant difference.

We first assessed the amounts of immune cell infiltration in the High MTRS and Low MTRS groups (**Figures 12A, B**). The immune cell types with notable abundance comprised M0 macrophages, CD4^+^ naïve T cells, M2 macrophages, and activated CD4^+^ memory T cells. The High MTRS group demonstrated a markedly elevated Tumor Immune Dysfunction and Exclusion (TIDE) score (**Figure 12C**), indicating enhanced immune suppression and an increased probability of unfavorable clinical outcomes in this cohort. Moreover, the Low MTRS group had a greater proportion of cell subpopulations lacking the immunological checkpoint markers CTLA-4 and PD-1, in contrast to their diminished presence in the High MTRS group (**Figure 12D**). The reduced expression of CTLA-4 and PD-1, pivotal immune checkpoint proteins linked to T cell exhaustion and immunological suppression, in the Low MTRS group may indicate a more active or less repressed immune condition. Consequently, in comparison to the High MTRS group, the Low MTRS group likely possesses a more vigorous antitumor immune response and a TME characterized by diminished immune suppression.

Among the five model genes, *XBP1*, *ETS2*, and *HEYL* exhibited increased CNV gains (**Figure 12E**). The High MTRS group consistently demonstrated increased ESTIMATE, Immune, and Stromal scores (**Figure 12F**). Correlation analyses demonstrated positive relationships between the risk prediction model and M0, M1, and M2 macrophage phenotypes, with notably robust positive correlations between *HEYL* and both M2 and M0 macrophages (**Figures 12G-I**). Additionally, the relationships among the five model genes, risk score, OS, and immune checkpoint-related genes were examined (**Figure 12J**). *HEYL* and *IRF7* exhibited a positive correlation with the majority of immunological checkpoint genes, whereas *ETS2* shown a negative correlation with most of these genes. Significantly, drug sensitivity analyses demonstrated that the High MTRS group displayed reduced half-maximal IC50 values for A.770041, AG.014699, WH.4.023, TW.37, Sunitinib, and GSK269962A, in comparison to the Low MTRS group, signifying heightened sensitivity to these compounds (**Figure 12K**). In contrast, Sorafenib and Mitomycin C exhibited reduced IC50 values in the Low MTRS group, indicating these medications may be more efficacious for patients within this subpopulation.

### Evaluation of enrichment in high- and low-risk categories

**Figure 13:**
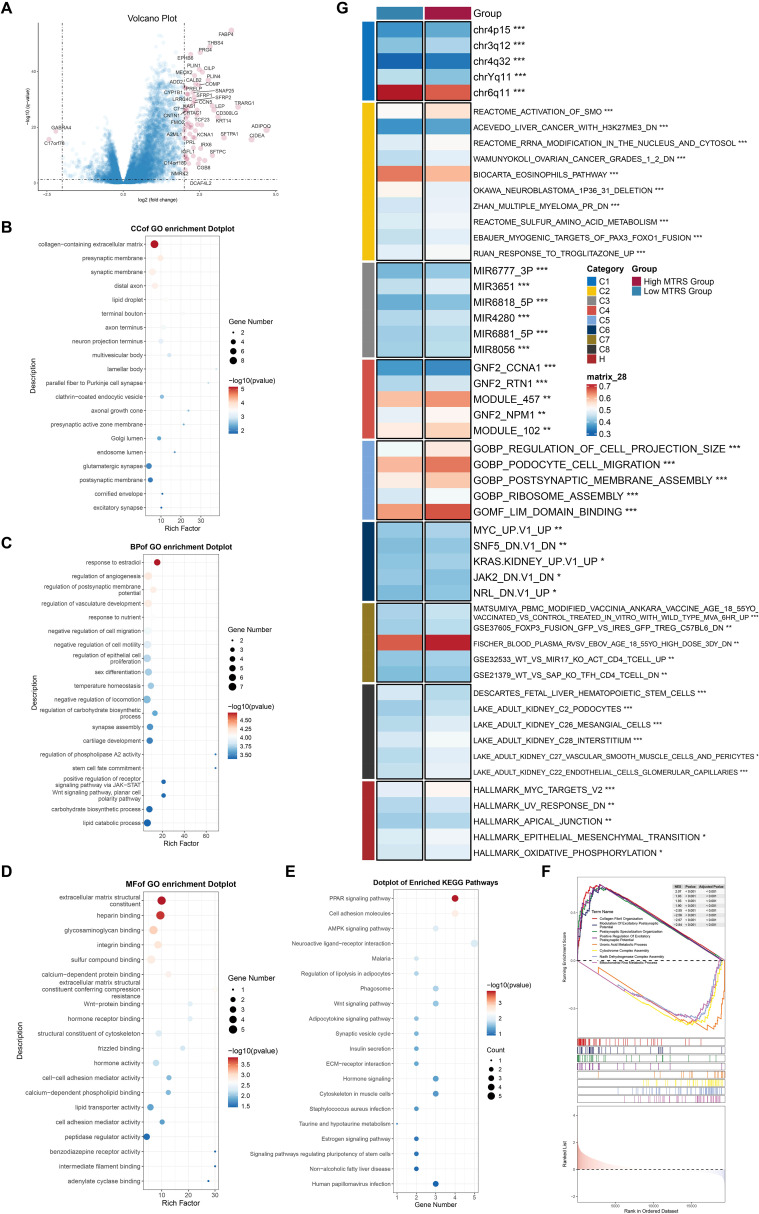
Enrichment analysis utilizing differentially expressed genes from high and low MTRS groups. **(A)** The volcano plot revealed significantly elevated and downregulated genes between the high and low MTRS groups. **(B–E)** Enrichment analysis for GO-CC, GO-BP, GO-MF, and KEGG pathways were conducted based on the differentially expressed genes between the two groups. **(F)** GSEA analysis was performed utilizing the differential gene sets between the high and low MTRS cohorts. **(G)** GSVA enrichment analysis yielded comprehensive results derived from the differential gene sets between the high and low MTRS cohorts. *P<0.05, **P<0.01, ***P<0.001.

We initially showed the DEGs between the high- and low-risk groups using a volcano plot, highlighting both upregulated and downregulated genes (**Figure 13A**). Subsequently, many enrichment analysis methodologies were employed, including Gene Ontology Cellular Component (GO-CC), GO-BP, Molecular Function (GO-MF), Kyoto Encyclopedia of Genes and Genomes (KEGG), and GSEA. In the GO-CC analysis, the upregulated genes were predominantly associated with the collagen-rich extracellular matrix, whereas the downregulated genes were primarily connected to the glutamatergic synapse and postsynaptic membrane (**Figure 13B**). In GO-BP, upregulated genes exhibited considerable enrichment in response to estradiol, whereas downregulated genes were concentrated in lipid catabolic and carbohydrate biosynthetic activities (**Figure 13C**). The GO-MF results demonstrated that elevated genes were primarily enriched in extracellular matrix structural components and heparin binding (**Figure 13D**). KEGG pathway analysis indicated that genes exhibiting elevated expression were predominantly associated with the *PPAR* signaling pathway, whereas genes demonstrating reduced expression were connected to pathways related to human papillomavirus infection (**Figure 13E**). GSEA corroborated these results, indicating that upregulated genes were considerably enriched in collagen fibril organization, positive control of excitatory postsynaptic potential, postsynaptic specialization organization, and modulation of excitatory postsynaptic potential. In contrast, downregulated genes exhibited enrichment in uronic acid metabolic processes, cytochrome complex assembly, NADH dehydrogenase complex assembly, and mitochondrial RNA metabolic processes (**Figure 13F**). Finally, GSVA was applied to gene sets from both the High and Low MTRS groups, with the outcomes illustrated in **Figure 13G**.

## Discussion

CRC continues to be a predominant cause of cancer-related mortality globally, representing over 10% of all newly diagnosed malignancies and cancer-related fatalities each year (1). Despite being classified as possibly curable, early-stage localized CRC still sees approximately 25% of patients diagnosed with metastatic disease at initial presentation, alongside others at risk for tumor recurrence, even with advancements in targeted therapies and cancer immunotherapies (41). Consequently, CRC remains a significant public health challenge, with an unfulfilled requirement for biomarkers to inform therapeutic strategies and prognostic assessments (42, 43). Additionally, CRC and CVD exhibit shared biology and risk variables (44, 45). Evidence indicates that individuals receiving therapy for CRC face an elevated long-term risk of developing CVD, partially attributable to the cardiotoxic effects of specific cancer medications (46). Chronic stress is a common risk factor for both CRC and CVD. It facilitates the development of an inflammatory TME and may hinder immunological responses by modifying immune cell functionality. Furthermore, prolonged stress can induce persistent inflammation, facilitating the progression of atherosclerosis and a prothrombotic condition (47–49). Consequently, CRC may be associated with detrimental cardiovascular outcomes through chronic inflammatory responses and immunosuppression within the TME (50). This study aimed to discover prospective targets for mitigating immunosuppression and inflammation in the CRC’s TME, while clarifying any links between CRC progression and cardiac dysfunction.

Initially, we picked CRC tumor tissues at various stages from pre-existing databases. EPCs demonstrated markedly elevated G2/M and S phase scores, along with more pronounced inferCNV signals. Stemness-related genes, including as *MYC* and *KLF4*, were significantly elevated in EPCs. Recent findings indicate that *PRMT6* facilitates CRC advancement by activating *MYC* signaling, whereas increased *MYC* activity and epithelial-mesenchymal transition (EMT) pathways may further intensify CRC malignancy (51, 52). *KLF4* has been demonstrated to generate CRC by facilitating epithelial-mesenchymal transition through *STAT3* activation (53). In addition to stemness characteristics, EPCs were chiefly linked to metabolic functions, particularly oxidative phosphorylation. Growing evidence indicates that the increase of oxidative phosphorylation facilitates the energy requirements of CRC growth and development (54). Furthermore, oxidative phosphorylation may facilitate cardiac fibrosis by activating cardiac fibroblasts, potentially leading to detrimental effects such as heart failure (55). *PINK1*, a gene frequently linked to EPCs, has been recognized as a tumor suppressor in CRC by modulating cellular metabolism. *PINK1* deficiency facilitates mitochondrial iron buildup and CRC development (56). Furthermore, EPCs were found to be abundant in pathways associated with cell adhesion and heart muscle contraction, possibly linking them to advanced CRC metastases and compromised heart function.

Subsequently, we discovered that across all tumor cell subpopulations, the C2 *MKI67^+^* TCs were mostly present in tumor stages II, III, and IV, called for their elevated expression of *MKI67*. *MKI67* has been identified as a potential diagnostic and prognostic biomarker for mismatch repair. -deficient/microsatellite instability-high colorectal carcinomas in stages II and III (57). The C2 *MKI67^+^* TCs demonstrated the highest G2/M and S phase scores across all subpopulations, accompanied by increased nFeature RNA and nCount RNA, indicating that these cells are highly proliferative and likely more malignant. We additionally evaluated differentiation potential among subpopulations utilizing CytoTRACE and CytoTRACE2 studies. The C2 *MKI67^+^* TCs exhibited the highest CytoTRACE scores, signifying a primarily undifferentiated or weakly differentiated state with substantial differentiation potential and, consequently, increased malignancy. This subpopulation comprised cells with several differentiation potentials, including differentiated, unipotent, oligopotent, and multipotent phenotypes, indicating a possibility for multilineage differentiation. Trajectory analysis utilizing Monocle positioned the C2 *MKI67^+^* TCs predominantly at the commencement of the developmental pathway, corroborating their undifferentiated status and implying that they may signify the initial phase of the cellular lineage or function as stem-like “source” cells. In the Slingshot-constructed Lineage 1, C2 *MKI67^+^* TCs exhibiting elevated CytoTRACE scores were situated at the terminal end, possibly indicating a distinct undifferentiated potential within the tumor differentiation trajectory. This subpopulation exhibited an enrichment of stemness-related genes, including *HMGA1*, as well as genes linked with migration and drug resistance, suggesting it may attain a “extreme undifferentiated” state through the EndMT pathway (58). These results suggest that the C2 *MKI67* ^+^ TCs subpopulation occupies a pivotal role in the TME and likely exerts a considerable influence on tumor invasion, metastasis, and chemotherapy resistance.

Alongside *MYC*, the C2 *MKI67* ^+^ TCs subpopulation had elevated expression of stemness-associated genes, including *EZH2*, *NOTCH1*, and *CD44*. *EZH2* is recognized as a significant biomarker in various malignancies, including colorectal and prostate cancer (59), and it has been shown to boost the growth and dissemination of CRC cells (60). A range of *EZH2* inhibitors has shown efficacy in inhibiting CRC via altering macrophage polarization in the TME (61). In CRC, the *NOTCH1*-mediated glycosylation-dependent Notch signaling pathway augments the stem-like characteristics of tumor cells, with *NOTCH*1 expression facilitating enhanced proliferation, migration, and invasion (62–64). *CD44* has been recognized as a predictive biomarker for immunotherapy efficacy in CRC and has a role in modulating macrophage polarization and tumor advancement (65). The upregulation of *CD44* enhances the proliferation and spread of CRC cells (66, 67). The heightened expression of these stemness-associated genes in the C2 *MKI67* ^+^ TCs subpopulation likely facilitates the aggressive progression of CRC. To ascertain whether the C2 *MKI67* ^+^ TCs subpopulation is a possible driver of CRC progression, we conducted enrichment analysis among tumor cell subpopulations. The five most upregulated genes in C2 *MKI67* ^+^ TCs were *NCAPH*, *TTK*, *NCAPG*, *ASF1B*, and *ARHGAP11A*, whereas the five most downregulated genes were *TSPAN1*, *FXYD3*, *NEAT1*, *LGALS4*, and *S100A6*. Integrative enrichment analyses repeatedly indicated that C2 *MKI67* ^+^ TCs are significantly linked to biological processes associated with cell proliferation and division, such as chromosome segregation, nuclear division, and organelle fission. These results support previous studies that used CytoTRACE and other methods, which strengthens the hypothesis that C2 *MKI67* ^+^ TCs have a greater ability to grow and divide. We argue that the C2 *MKI67* ^+^ TCs subpopulation is a crucial cell subpopulation for examining the cellular landscape of CRC progression and presents a promising target for possible treatment methods.

Throughout the onset and advancement of CRC, tumor cells that serve pivotal roles are very likely to interact with many other cell types. Tumor cells can promote the polarization of remote immune cells, such as macrophages, through exosomes, so affecting the immunological milieu of the heart (68–70). In immunology, TAMs can be divided into two groups based on how active they are and what they do: M1 type and M2 type. Interferon-γ (IFN-γ), lipopolysaccharide (LPS), and other things can stimulate and activate M1-type macrophages. They can show antigens well and cause inflammation, and they can boost the immune system’s ability to fight tumors by making things like TNF-α and IL-12. On the other hand, M2-type macrophages are frequently triggered by signals like IL-4, IL-10, and TGF-β. They then do things like suppress the immune system, promote angiogenesis, and help tumors grow and change tissues (71, 72). Furthermore, Advanced CRC is also often connected to immunological suppression or dysregulation. This is shown by tumor-associated macrophages that are polarized to the M2 phenotype, T cell exhaustion, and higher levels of systemic inflammatory mediators (73). These immunological modifications lead to increased myocardial fibrosis, cardiomyocyte damage, and diminished heart functional capacity. Patients with CRC often suffer from cancer cachexia, a syndrome that may result in myocardial atrophy, cardiac dysfunction, and structural remodeling (74, 75). In this context, we illustrated the interaction between the principal subpopulation C2 *MKI67* ^+^ TCs and macrophages via CellChat. A substantial interaction is seen, principally mediated by the MIF-(CD74+CD44) signaling axis. Previous studies have shown that when CD74 and CD44 are both present in CRC, the tumor becomes more aggressive (76). Additionally, RNA m6A methylation-associated cellular subpopulations within the TME and tumor epithelial cells can participate in many and comprehensive interactions through ligand-receptor pairings, such as MIF-(CD74+CD44) (77). The MIF-(CD74+CD44) axis is present and essential in CRC, suggesting that C2 *MKI67* ^+^ TCs may affect CRC progression and maybe late-stage cardiac function via this pathway, necessitating additional research. Therefore, We found the MIF-(CD74+CD44) signaling axis to be a pathway that hasn’t been studied as much as the TGF-β and IL-1 pathways (78, 79). However, it has a lot of potential for regulating the immune system, especially because it has a strong paracrine effect in the one-way communication between C2 *MKI67^+^* TCs and macrophages. In the immunological microenvironment of colorectal cancer, this pathway may work on its own or with the classical system. This gives us a fresh notion for a combination immunotherapy strategy that targets both the traditional and new pathways. Furthermore, we want to stress that the varied ways that cells communicate with each other in this study, especially the ligand-receptor interaction between tumor cells and immune cells, give us fresh ideas for how to understand why immunotherapy works differently for different people. For example, if there are active immune activation signals between some tumor subpopulations and T cells (such CD80/CD28 or IFNG/IFNGR) (80), it could mean that the patient is more likely to respond to immunity therapy and could benefit from immune checkpoint inhibitors. But if the major way cells talk to one other is through immunosuppressive pathways like CD47/SIRPA and TGFB1/TGFBR1, it could mean that the cells are likely to become resistant to drugs (81). So, studying cellular communication networks in depth not only helps us understand how they work, but it could also lead to the discovery of biomarkers that can predict how well immunotherapy will work, which will be very useful in clinical practice in the future.

To further investigate the transcriptome attributes of the important subpopulation, we conducted a visualization analysis of C2 *MKI67* ^+^ TCs. Within these cells, five TFs—*E2F8*, *E2F1*, *E2F7*, *HMGA1*, and *BPTF*—exhibited elevated expression levels. Our prior findings using CytoTRACE and Slingshot indicated that C2 *MKI67* ^+^ TCs may express genes such *HMGA1*, which could facilitate a “extremely undifferentiated” state through the EndMT process. This theory is corroborated by their elevated CytoTRACE scores and terminal placement on the Slingshot trajectory. The depiction of the transcriptional regulatory landscape corroborates and elucidates this observation. The *E2F* family of transcription factors is recognized for its role in controlling different cellular activities associated with the cell cycle and programmed cell death (82). Several studies have shown that *E2F8* plays a crucial role in controlling cell growth, differentiation, and programmed cell death (83, 84). *E2F8* is increased in CRC tissues, influencing the expression of cell cycle genes; additionally, *E2F8* inhibition attenuates CRC cell proliferation through the *NF-κB* pathway (85, 86). *E2F7* promotes the proliferation, motility, and invasion of CRC cells and enhances the functionality of CRC tumor stem cells (87, 88), while *E2F1* exhibits tumor-suppressive functions in CRC (89). In addition to the *E2F* family, *HMGA1* plays a role in CRC carcinogenesis by enhancing lipid synthesis, since research indicates that *HMGA1* overexpression promotes the migration and invasion of CRC cells (90, 91). Utilizing the CSI matrix, we categorized TFs among all tumor cell subpopulations into three regulatory modules. Module M3, characterized by C2 *MKI67* ^+^ TCs, has elevated transcriptional activity of *E2F8*, *E2F1*, and *HMGA1*, corroborating prior findings and reinforcing the role of these factors as pivotal transcriptional regulators within this subpopulation. Although the *E2F* family has been extensively studied as possible therapeutic targets in many malignancies, research on *HMGA1* is rather scarce despite its acknowledged significance in advancing CRC progression. We propose that *HMGA1* may function as a novel therapeutic target for the treatment of CRC. To ascertain *HMGA1*’s function, we performed a series of *in vitro* tests. *HMGA1* was silenced in two CRC cell lines, HCT116 and HT-29. Colony formation assays, EDU labeling, and wound healing experiments consistently indicated that *HMGA1* knockdown markedly suppressed CRC cell growth. Furthermore, wound healing and Transwell experiments demonstrated significant inhibition of CRC cell migration and invasion subsequent to *HMGA1* knockdown. The data suggest that the TF *HMGA1*, which is significantly expressed in C2 *MKI67* ^+^ TCs, likely facilitates CRC progression. It is also worth noting the necessity of conducting *in vivo* experiments in the future, including relevant experimental plans such as tumor transplantation models, to verify more comprehensively.

Simultaneously, we aimed to investigate the possible association between CRC and the deterioration of cardiac function by utilizing the discovered critical subpopulation, C2 *MKI67* ^+^ TCs. We assembled a gene collection linked to heart failure and cardiac fibrosis by gathering genes that are expressed in CRC tumor tissues and are strongly correlated with cardiac dysfunctions, including heart failure and myocardial fibrosis, based on rigorous prior research and established consensus. Utilizing this gene set, we evaluated all tumor cell subpopulations and discovered that C2 *MKI67* ^+^ TCs—predominantly in the G2/M and S phases—demonstrated significantly heightened heart failure scores, whereas their cardiac fibrosis scores were not substantially elevated. It should be emphasized here that this score does not directly reflect “actual cardiac function”, but is constructed based on the correlation of gene expression related to heart failure. Therefore, this indicates that this subpopulation may engage non-fibrotic pathways associated with heart failure, like mitochondrial malfunction, metabolic imbalance, or oxidative stress. Alternatively, it may suggest that C2 *MKI67* ^+^ TCs contribute indirectly to the advancement of heart failure by the production of factors or exosomes, rather than acting as primary instigators of myocardial fibrosis (92). Our past research on cell communication showed that there was a lot of crosstalk between the C2 subpopulation and M2 macrophages, which supports the second theory. We need to go into the processes behind this more.

We should also point out that the current evidence mostly comes from indirect inferences of gene expression patterns. However, our analysis of risk scores and immunosuppression status has shown that the C2 *MKI67* ^+^ TCs subpopulation plays an important role in promoting tumor inflammatory responses and immunosuppression and has a high “heart failure score.” There is still no apparent cause-and-effect relationship. So, future studies might look into making a more full validation framework. For example, they could build CRC *in situ* or metastatic animal models and use myocardial tissue transcriptome/histopathological analysis to see how tumor load affects distal cardiac performance. Keep an eye on how important things in tumor-derived exosomes move about, including whether inflammatory substances like MIF and IL-6 can get across the vascular barrier and affect cardiomyocytes or cardiac immune cells, and so on.

This study seeks to offer potential prognostic and therapeutic intervention techniques for CRC patients. Consequently, we developed a risk prediction model centered on the pivotal subpopulation C2 *MKI67* ^+^ TCs. In the High MTRS group, risk genes including *HEYL*, *MAFK*, and *IRF7* displayed increased expression, with the *HEYL*-associated gene signature showing strong predictive capability (93). Genes exhibiting differential upregulation between high- and low-risk groups were mostly linked to pathways involving extracellular matrix structural components and collagen fibril organization. We performed a comprehensive investigation of immune infiltration disparities between the two risk groups, observing elevated numbers of M0 macrophages, CD4^+^ naïve T cells, M2 macrophages, and activated CD4^+^ memory T cells. This may signify a shift in the immune microenvironment from inflammation to repair or demonstrate tumor-associated immune evasion. The simultaneous occurrence of M0 and M2 macrophage phenotypes likely indicates tumor-promoting signals in the microenvironment (94–96). M0, M1, and M2 macrophage profiles had a favorable correlation with the risk model, with M2 and M0 macrophages demonstrating greater relationships with *HEYL* expression. The significant increase of CD4^+^ naïve T cells may indicate compromised antigen presentation or an immunosuppressive environment that obstructs early T cell activation, thereby undermining effective anti-tumor immunity. The enhancement of activated CD4^+^ memory T cells may stem from previous immunological responses to tumor antigens, with their activated condition potentially supporting anti-tumor immunity. The makeup of immune cells suggests a TME characterized by partial immunological activation and overall immune suppression, offering insights into mechanisms of tumor immune evasion and informing the development of immunotherapies (97, 98). Furthermore, Many different types of tumors have used the TIDE and ESTIMATE immune scoring systems to estimate how well immunotherapy will work. The first one simulates the immune escape mechanism to check how well T-cells work. The second one can figure out how pure a tumor is and how many immune and interstitial cells are in it. So, it is quite useful for checking the health of the immunological microenvironment and the possible benefits of treatment. Thus, the High MTRS group displayed increased TIDE scores, indicating greater immunological suppression (99). The immune checkpoint molecules CTLA-4 and PD-1, recognized indicators of T cell exhaustion and immune suppression, exhibited elevated expression in the High MTRS group, whereas numerous cells in the Low MTRS group demonstrated low or negligible expression, suggesting a relatively more active anti-tumor immune response or a less immunosuppressive TME in the Low MTRS group (100). Moreover, *HEYL* and *IRF7* exhibited a favorable correlation with the majority of immune checkpoint-related genes, thereby reinforcing these findings. Of course, in the future, the generalization ability of this model can be verified by combining spatial omics or clinical imaging data to enhance its practical value and clinical application prospects.

Alongside the previously mentioned risk and prognosis evaluations, we performed pharmacological sensitivity analysis to investigate possible therapy approaches. We want to provide insights for future CRC therapy alternatives by emphasizing the disparities in sensitivity to different immunotherapeutic drugs across high-risk and low-risk populations. Our results indicate that the High MTRS group exhibits increased sensitivity to A.770041, AG.014699, WH.4.023, TW.37, Sunitinib, and GSK269962A. A.770041 is an investigational small chemical that selectively inhibits Lck (lymphocyte-specific protein tyrosine kinase), potentially regulating T cell activation and function (101, 102). AG.014699, referred to as Rucaparib, is a PARP (poly ADP-ribose polymerase) inhibitor sanctioned for the treatment of ovarian, breast, pancreatic, and prostate malignancies (103, 104). WH.4.023 is an investigational small molecule inhibitor of ROCK (Rho-associated protein kinase); research indicates that WH.4.023 is efficacious against a demethylated CRC subtype marked by immune suppression and downregulation of critical immunological pathways (105). TW.37 is a BH3 mimetic small chemical that specifically targets Bcl-2 family proteins to induce apoptosis in cancer cells, demonstrating efficacy in pancreatic, ovarian, and neuroblastoma malignancies (106–109). Sunitinib is an orally taken multi-target receptor tyrosine kinase inhibitor that can reverse immune suppression, making it a prospective adjuvant to improve the efficacy of immunotherapy in advanced cancers (110). Numerous studies have indicated Sunitinib’s efficacy in combination therapy for CRC (111–114). GSK269962A is a selective inhibitor of ROCK, largely utilized to examine the functions of ROCK in cell migration, proliferation, and angiogenesis (115). Sunitinib has received clinical approval for several malignancies, whilst the other drugs are predominantly in the experimental phase. Consequently, our findings may yield significant insights for the therapeutic utilization of these drugs and serve as prospective references for future CRC immunotherapy approaches.

This study integrates single-cell analysis, *in vitro* experiments, and the development of cancer prediction models to thoroughly characterize the C2 *MKI67* ^+^ TCs subpopulation, thereby enhancing prospects for early CRC screening, prognostic risk evaluation, and targeted therapy, including the identification of potential biomarkers and therapeutic targets. We meticulously analyzed the intricate regulatory network inside the TME of colorectal cancer, namely the immune signaling interactions between tumor cells and macrophages via the MIF-(CD74+CD44) signaling pathway. The C2 *MKI67* ^+^ TCs subpopulation may have a potential association with CRC inflammation and cardiac dysfunction resulting from immunosuppression. Consequently, the *MKI67* ^+^ TCs subpopulation may contribute to immune signaling and enhance inflammatory responses inside TME of potential colorectal cancer, hence facilitating the identification of novel immunotherapy targets. However, certain limits must be acknowledged. The restricted sample size of the data set may constrain the generalizability of our findings. Future research with bigger multicenter cohorts will be crucial to validate the proposed prognostic model and the universality of the identified targets. Secondly, our experimental validation was confined to preliminary *in vitro* investigations focused on *HMGA1*. Further comprehensive *in vivo* and *in vitro* investigations are required to clarify its precise function in CRC. Third, while we originally evaluated the gene sets linked to heart failure and myocardial fibrosis across different tumor cell subpopulations, the gene sets employed may not fully encompass the genes intrinsically connected with colorectal cancer and cardiac dysfunction. Consequently, the accuracy of these scores may be constrained. The potential immunosuppressive impact of advanced colorectal cancer on heart function is an underexplored domain necessitating further mechanistic investigations. Furthermore, there is no geographical transcriptome data to back up this study. The assumed patterns of communication between cells are mostly based on the probability model of ligand-receptor co-expression. However, it is still not able to explicitly prove the spatial closeness and co-localization relationship of these cells in the tissue microenvironment. So, future studies need to use spatial omics technologies (like 10x Genomics Visium, MERFISH, or spatial proteomics) to confirm the existence and active regions of key signal axes from the spatial dimension and to make clear the real spatial organizational structure and functional interaction relationship between certain cell subpopulations. In conclusion, we believe that our research offers significant insights and potential avenues for CRC intervention and personalized therapy. We are dedicated to addressing these limitations in future studies to achieve more definitive and significant conclusions.

## Conclusion

Our study identified the C2 *MKI67* ^+^ TCs subpopulation as a key driver of immune signaling and inflammation within the CRC TME. Predominantly enriched in advanced CRC and characterized by high proliferative and poorly differentiated features, this subpopulation is associated with poor prognosis. C2 *MKI67* ^+^ TCs may interact with immune cells, particularly macrophages, through distinct signaling pathways, contributing to immunosuppression in the TME. Notably, this subpopulation also showed a significant heart failure score, suggesting a potential link to inflammation-driven cardiac dysfunction in CRC. Transcriptionally, we identified *HMGA1* as a critical regulator with potential as an immunotherapy target, and its functional role was preliminarily validated *in vitro*. Finally, we constructed a cancer risk prediction model based on this subpopulation to support individualized CRC treatment strategies. Future work will aim to address current limitations and further elucidate the mechanisms underlying immune modulation and disease progression.

## Data Availability

The original contributions presented in the study are included in the article/**Supplementary Material**. Further inquiries can be directed to the corresponding authors.

## Supplementary material

Supplementary Figure 1 The analysis of inferCNV. **(A)** InferCNV analysis utilizing scRNA-seq data from ECs was conducted to forecast copy number variations, with red denoting amplifications and blue signifying deletions.
