# Supplementary figures and images for: Single-cell technology reveals the crosstalk between tumor cells and immune cells: driving immune signal transduction and inflammation-mediated cardiac dysfunction in the tumor microenvironment of colorectal cancer
Source: Front Immunol. 2025 Aug 6;16:1637144. doi: 10.3389/fimmu.2025.1637144 (PMC12364672; doi:10.3389/fimmu.2025.1637144)

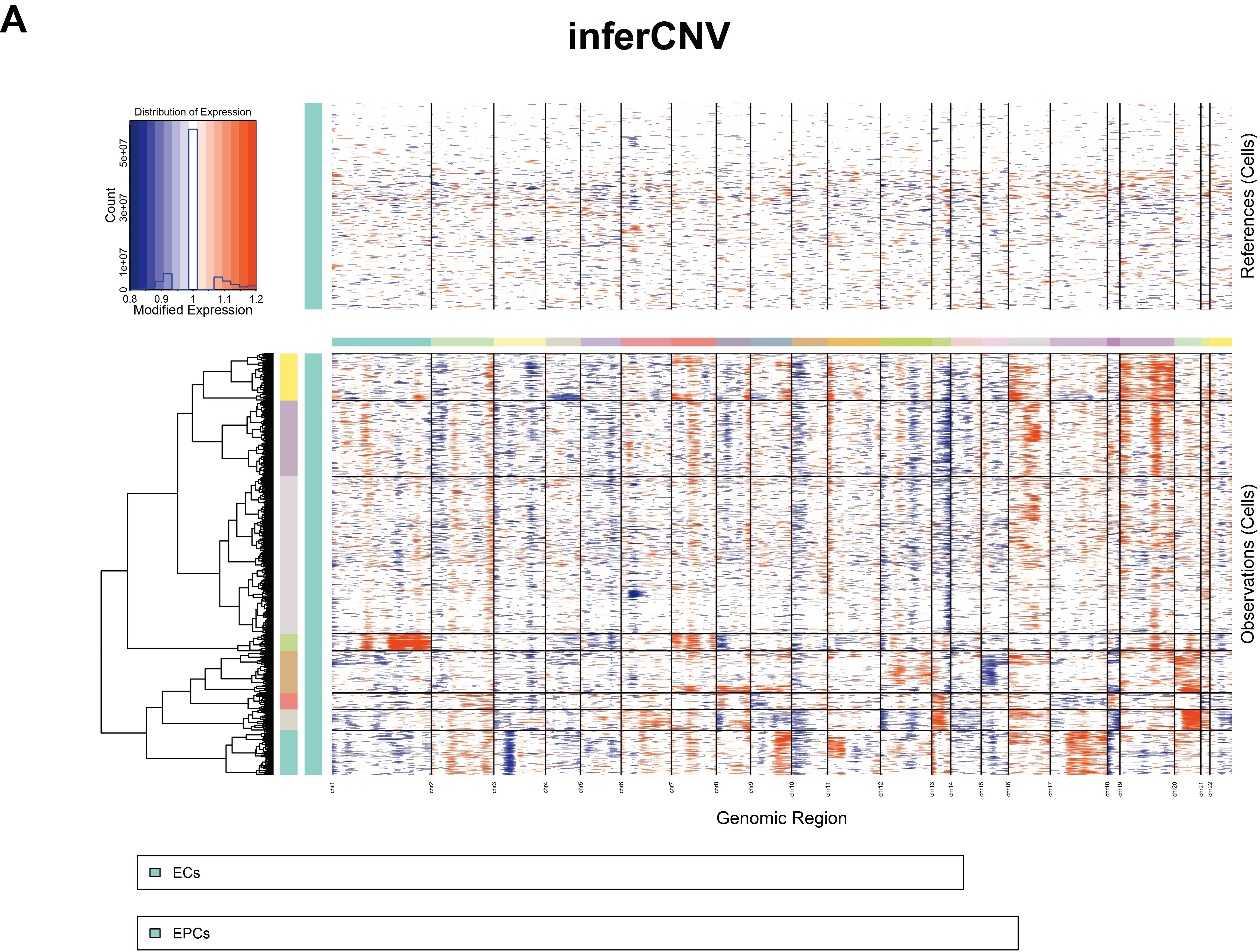

Supplement: Supplementary Figure 1 — The analysis of inferCNV. (A) InferCNV analysis utilizing scRNA-seq data from ECs was conducted to forecast copy number variations, with red denoting amplifications and blue signifying deletions. [file Image1.jpeg]
